# Sucrose preferentially promotes expression of *OsWRKY7* and *OsPR10a* to enhance defense response to blast fungus in rice

**DOI:** 10.3389/fpls.2023.1117023

**Published:** 2023-01-27

**Authors:** Win Tun, Jinmi Yoon, Kieu Thi Xuan Vo, Lae-Hyeon Cho, Trung Viet Hoang, Xin Peng, Eui-Jung Kim, Kay Tha Ye Soe Win, Sang-Won Lee, Ki-Hong Jung, Jong-Seong Jeon, Gynheung An

**Affiliations:** ^1^ Graduate School of Green-Bio Science, Kyung Hee University, Yongin, Republic of Korea; ^2^ Department of Plant Bioscience, Pusan National University, Miryang, Republic of Korea; ^3^ Rice Research Institute, Guangdong Academy of Agricultural Sciences, Guangzhou, China

**Keywords:** *Magnaporthe oryzae*, OsPR10a, OsWRKY7, rice, sucrose

## Abstract

Sucrose controls various developmental and metabolic processes in plants. It also functions as a signaling molecule in the synthesis of carbohydrates, storage proteins, and anthocyanins, as well as in floral induction and defense response. We found that sucrose preferentially induced *OsWRKY7*, whereas other sugars (such as mannitol, glucose, fructose, galactose, and maltose) did not have the same effect. A hexokinase inhibitor mannoheptulose did not block the effect of sucrose, which is consequently thought to function directly. MG132 inhibited sucrose induction, suggesting that a repressor upstream of *OsWRKY7* is degraded by the 26S proteasome pathway. The 3-kb promoter sequence of *OsWRKY7* was preferentially induced by sucrose in the luciferase system. Knockout mutants of *OsWRKY7* were more sensitive to the rice blast fungus *Magnaporthe oryzae*, whereas the overexpression of *OsWRKY7* enhanced the resistance, indicating that this gene is a positive regulator in the plant defense against this pathogen. The luciferase activity driven by the *OsPR10a* promoter was induced by OsWRKY7 and this transcription factor bound to the promoter region of *OsPR10a*, suggesting that OsWRKY7 directly controls the expression of *OsPR10a*. We conclude that sucrose promotes the transcript level of *OsWRKY7*, thereby increasing the expression of *OsPR10a* for the defense response in rice.

## 1 Introduction

Sugars derived from the photosynthetic process are the basic raw materials for cell vitality and function as intermediate substances to build macromolecules such as nucleic acids and the cell wall (Plaxton, 1996; Wang et al., 2007). Sugars also act as signal molecules in various cellular processes, such as seed germination, seedling growth, storage organ development, flowering, and senescence (Li and Sheen, 2016; Sakr et al., 2018; Yoon et al., 2021). Among various sugars, glucose is widely accepted as a signaling molecule in plants, yeast, and mammals. However, studies on sucrose-specific signaling are scarce, because this sugar is readily metabolized to fructose and glucose in plants. It has been shown that a significant amount of glucose is converted to sucrose in detached tobacco leaves within 8 hours after glucose feeding (Morcuende et al., 1998). Similarly, nourishing detached spinach leaves with glucose rapidly increases their sucrose level (Krapp et al., 1991).

Nonetheless, several studies have also reported that the transcript levels of various genes are preferentially controlled by sucrose (Yoon et al., 2021). For example, the mRNA level of the sucrose transporter *BvSUT1* is reduced by a high concentration of sucrose in sweet beet leaves (Nieberl et al., 2017). Sucrose also preferentially induces various genes that encode starch biosynthesis enzymes, such as UDP-glucose pyrophosphorylase, ADP-glucose pyrophosphorylase (AGPase), and granule-bound starch synthase (Müller-Röber et al., 1992; Ciereszko et al., 2001; Wang et al., 2001; Li et al., 2002).

Sucrose functions as a specific signaling molecule to control plant development stages (Yoon et al., 2021). *Arabidopsis* plants that overexpress the homeodomain–leucine zipper gene, *ATH13*, show the narrow leaf phenotype when they are grown on sucrose-containing medium (Hanson et al., 2001). Sucrose concentration in the phloem sap increases dramatically upon floral induction in various plant species, such as *Sinapis alba, Xanthium strumarium, Lolium temulentum*, and *Arabidopsis thaliana* (Bodson and Outlaw, 1985; Houssa et al., 1991; Corbesier et al., 1998). Exogenous application of sucrose promotes flowering in *Brassica campestris, S. alba*, and chrysanthemum (Friend et al., 1984; Sun et al., 2017).

Sucrose also functions as a signaling molecule to protect plants from pathogen invasion by modulating the production of peroxidase and pathogen-related proteins (Thibaud et al., 2004; Morkunas et al., 2005). The expression of the maize fungal-inducible *PRms* gene in transgenic tobacco and rice plants causes the accumulation of a high level of sucrose that provides protection against various pathogens, which suggests that this sugar may promote plant defense (Murillo et al., 2003; Gómez-Ariza et al., 2007). In line with this hypothesis, the exogenous application of sucrose was shown to induce the expression of several PR genes in tobacco and rice (Murillo et al., 2003; Gómez-Ariza et al., 2007). Similarly, sucrose was reported to induce the expression of genes *PR-1*, *PR-2*, and *PR-5* in *Arabidopsis* (Thibaud et al., 2004). Although glucose also increases the *PR-2* transcript level, the induction is weak and non-metabolizable glucose analogs (i.e., 3-*O*-methylglucose and 2-deoxyglucose) do not promote gene expression, suggesting that the induction is not hexokinase dependent.

Several WRKY transcription factors play a role in the defense response in various plant species, including *Arabidopsis*, rice, wheat, grape, pepper, and cotton (Jiang et al., 2016; Jimmy and Babu, 2019). In rice, several WRKY genes are rapidly induced or repressed upon infection with bacterial and fungal pathogens, such as *Xanthomonas oryzae* pv. *oryzae* (*Xoo*) and *Magnaporthe oryzae*, respectively (Ryu et al., 2006; Jimmy and Babu, 2015). Among these genes, more than 10 were shown to induce resistance against fungal blast (Chen and Wang, 2014). For example, *OsWRKY22* appears to be involved in defense responses because the *oswrky22* mutant lines are highly susceptible to *M. oryzae* and *Blumeria graminis* (Abbruscato et al., 2012). Another WRKY gene, *OsWRKY30*, which is inducible by salicylic acid (SA) and jasmonic acid (JA), also enhances resistance to *M. oryzae*, *Rhizoctonia solani*, and *Xoo* when it is overexpressed in transgenic rice plants (Peng et al., 2012). Tao et al. (2009) identified a pair of *OsWRKY45* alleles that function as a positive factor during *M. oryzae* infections but play an opposite role in the defense against bacterial pathogens. Whereas *OsWRKY45-1* knockout plants are resistant to *Xoo* and *X. oryzae* pv. *oryzicola*, *OsWRKY45-2*-suppressing plants show increased susceptibility to these pathogens. OsWRKY45 forms a heterodimer with OsWRKY62 that acts as a strong activator, while the OsWRKY62 homodimer acts as a repressor (Fukushima et al., 2016). A number of WRKY factors function negatively in defending the plants against *M. oryzae*. The transcriptional repressor OsWRKY13 binds to the promoter regions of both the *OsWRKY45-1* and *OsWRKY45-2* genes, thereby repressing the target gene expression (Xiao et al., 2013). Similarly, OsWRKY42, which functions downstream of WRKY45-2, negatively affects rice resistance by suppressing JA-dependent signaling (Cheng et al., 2015).

Sucrose induces several WRKY genes. For example, it was shown to increase the mRNA level of *AtWRKY20*, which activates the *AGPase* gene in *Arabidopsis* (Nagata et al., 2012). Similarly, sucrose addition to *Arabidopsis* leaves induces the ectopic expression of an endosperm-specific WRKY transcription factor, *SUSIBA2* (Sun et al., 2003). However, the effects of other sugars have not been examined in either of the two above-mentioned studies. In the present study, we investigated the signal roles of sucrose in plant defense.

## 2 Materials and methods

### 2.1 Plant materials and growth conditions

The Oc suspension cell line that was established from seedling roots of *Oryza sativa* L. indica type accession C5928 (Baba et al., 1986) was cultured in 50 mL of R2S liquid medium (Ohira et al., 1973) in a 120-rpm shaking incubator at 28°C, as previously reported (Cho et al., 2016; Yoon et al., 2021). After 2 weeks of culture, 20 mL of the upper liquid layer was decanted, and 10 mL of the remaining cell suspension was transferred to a fresh flask containing 40 mL of R2S culture medium.

The *OsWRKY7* mutant (line number 5A-00022) was identified from T-DNA tagging lines generated in *Oryza sativa* var. *japonica*, cultivar ‘Dongjin’ (Jeon et al., 2000; Jeong et al., 2002; An et al., 2005a; An et al., 2005b; Jeong et al., 2006). Homozygous mutant plants from the T2 progeny were identified by PCR using genomic DNA isolated from the blade of the second leaf of each plant. The genotyping primers were GTGTCGGGTGCGTATTTAAAAAC (F), GGGAATTGGCGCTAAATCTGC (R), and atccagactgaatgcccacag (NGUS2) (**Supplementary Table 1**). Null mutations of *OsWRKY7* were obtained using the CRISPR/Cas9 method in ‘Donjin’ background. Homozygous mutants at T1 generation were detected through the subcloning and multiple sequencing of the mutated region using the genomic DNA extracted from the leaf blades of individual plants.

For overexpression of *OsWRKY7*, a full length of the gene was cloned by PCR using the specific primers (**Supplementary Table 1**) with *Hind*III and *Sac*I restriction enzyme sites. The gene was subcloned between the maize ubiquitin (*ZmUbi*) promoter and HA tag in the binary vector pGA3428 (Kim et al., 2009). The resulting vector was used to generate transgenic rice plants overexpressing OsWRKY7-HA. Produced plants were verified by western blot analysis using protein extracts from the leaf blades after 24 hours of *M. oryzae* infection. All plants were grown in the paddy field or in a controlled growth room under a long day conditions (i.e., 14 hours of light at 28°C/10 hours of darkness at 23°C, humidity of approximately 50%).

### 2.2 RNA-sequencing and data analysis

Oc cells were grown for 10 days in R2S solution complemented with 3% glucose as a carbon source. They were divided into two equal parts; one was added with sucrose and the other with glucose, obtaining a final sugar concentration of 3% in two replications. Samples were collected immediately before the transfer to new media (0 h) and 4 hours after sugar addition (4 h). Total RNA was extracted using the QIAGEN RNA purification kit (Hilden, Germany), and the purity and concentration of RNA were estimated using a NanoDrop 2000 spectrophotometer (Thermo scientific). RNA-seq analysis was performed based on protocols previously reported in Peng et al. (2021). The gene ontology (GO) terms of the differentially expressed genes were analyzed using the Rice Oligo Array Database (ricephylogenomics-khu.org). All the mapped genes were downloaded and filtered based on the following criteria: hyper *p*-value < 0.05 and query numbers > 10. Additional significance of the GO terms was denoted by a fold enrichment value > 1.5, which was obtained by dividing the query number by the query expectation value (Hong et al., 2020; Kim et al., 2021). The biotic stress category was obtained *via* the MapMan analysis toolkit 3.5.1 R2 (Thimm et al., 2004).

### 2.3 RNA isolation and the expression analysis

Total RNA was extracted from the leaves, roots, and Oc cells using RNAiso Plus (Takara, Japan). After combining 3 µg of RNA with 10 ng of oligo (dT), the first-strand cDNA was synthesized using 100 units of Moloney murine leukemia virus (M-MLV) reverse transcriptase (Promega, Madison, WI, USA), 100 units of RNasin (Promega), and 2.5 mM deoxyribonucleotide triphosphates. The gene expression levels were analyzed by quantitative RT-PCR using SYBR Green I, Prime Q-Matermix (2x) (GENET Bio, Daejeon, Korea) in a Rotor-Gene Q system (Qiagen, Hilden, Germany). Rice *Actin1* (*OsActin1*) was used as an endogenous control to normalize the expression level of the respective genes. All experiments were repeated at least three times, and the relative transcript levels were calculated using the ΔΔCt method (Schmittgen and Livak, 2008; Yoon et al., 2022). The primers used in this study are listed in **Supplementary Table 1**.

### 2.4 Generation of CRISPR/Cas9 mutants

After selecting a potential target sequence with the CRISPR web tool, the RNA scaffold structure was analyzed using the RNAfold web server (univie.ac.at), and off-target sites were checked with the web tools (http://crispr.dbcls.jp; Naito et al., 2015 and CRISPOR (tefor.net); Concordet and Haeussler, 2018). A potential target sequence (5’-ATGTCATCGTACTTCTCCCA-3’) within the first exon of *OsWRKY7* was cloned into the CRISPR vector pRGEB32 through the *Bsa*I restriction site. The construct was transformed into the *Agrobacterium tumefaciens* strain LBA4404 using the freeze–thaw method (An, 1987). Transgenic rice plants were obtained *via* the *Agrobacterium*-mediated co-cultivation method (Lee et al., 1999).

### 2.5 Plasmid construction for the reporter assay

The 3.081-kb promoter region (−3098 to −18 bp from the ATG start codon) of *OsWRKY7* from cultivar ‘Nipponbare’ was cloned into the *Hind*III-digested pGA3452 vector (Kim et al., 2009) upstream of the luciferase (*LUC*) coding region using the 5X In-Fusion HD enzyme Premix (Takara, Japan), which generated the p*OsWRKY7-LUC* reporter vector. The 2.856-kb promoter portion (−2856 to −1 bp from the start ATG codon) of *OsPR10a* was inserted into pGA3452, which resulted in p*OsPR10a-LUC*. The *ZmUbi-GUS* (Cho et al., 2009) construct was used as the internal control. For effector constructs, the coding regions of *OsWRKY7, OsWRKY28*, and *OsWRKY10* were amplified by PCR and were inserted between the *ZmUbi* promoter and 6X Myc of pGA3697 (Kim et al., 2009).

### 2.6 Transient assay

Protoplasts were prepared from Oc cells based on methods described in Cho et al. (2016) and He et al. (2016). In brief, Oc cells were incubated in an enzyme solution (2% cellulose RS, 1% macerozyme, 0.1% CaCl_2_, 0.4 M mannitol, 0.1% MES, pH 5.7) for 4 hours at 28°C in the dark. After incubation, they were added with an equal volume of KMC solution (117 mM potassium chloride, 82 mM magnesium chloride, and 85 mM calcium chloride). The number of protoplasts was quantified with a hemocytometer to ensure that a cell density of 2 × 10^6^ cells mL^−1^ was reached. The isolated protoplasts were transformed with 10 µg of each plasmid DNA using the PEG method (Yoo et al., 2007; Cho et al., 2022). *ZmUbi : GUS* construct (2 µg) was co-transfected as an internal control (Cho et al., 2009). The GUS activity was used to normalize the LUC activity.

### 2.7 Chromatin immunoprecipitation assay

The chromatin immunoprecipitation (ChIP) assay was performed using the protocols described in Haring et al. (2007) and Yoon et al. (2022) with some modifications. Firstly, the fresh leaves from the *OsWRKY7-HA* transgenic plants were collected and the proteins and chromatin were cross-linked using 3% formaldehyde. After the isolation of nuclei, DNA was broken into fragments of approximately 200–800 bp in length by sonication. Agarose beads of G and A proteins (Milipore; http://www.emdmilipore.com) were used to preclear the sheared chromatin for 1 hour at 4°C. Anti-HA monoclonal antibody (C29F4, https://www.cellsignal.com) was used for immunoprecipitation together with the precleared agarose beads for 4 hours at 4°C. The washing and reverse cross-linking steps described in Haring et al. (2007) were followed. The enriched chromatin was analyzed by qRT-PCR using the primers listed in **Supplementary Table 1**. The obtained data were normalized using the fold enrichment method (Haring et al., 2007; Yoon et al., 2022).

### 2.8 Electrophoresis mobility shift assay (EMSA)

The oligonucleotide (-808 to -831 from the start ATG of *OsPR10a*) was end labeled with biotin (Microgen, Korea). To produce MBP-tagged OsWRKY7 protein, the coding sequence of *OsWRKY7* was amplified by PCR and cloned into the pMAL-c2X vector (Addgene plasmid # 75286; http://n2t.net/addgene:75286; RRID : Addgene_75286; walker et al., 2010) through the *EcoR*1 and *Sal*1 enzyme sites. The MBP-OsWRKY7 protein were extracted using amylose resin (E8021S, New England, Biolabs Inc.) The MBP-OsWRKY7 protein (1 µg) and biotin labeled ds-DNA fragment (750 fmol) were incubated in the reagent (KIT 0020148, Thermo scientific) containing 1× binding buffer, 10% glycerol, 5 mM MgCl_2_, 0.5 µg poly (dI-dC), and 0.05% NP-40 in a final volume of 20 µL for 30 min at 25°C. After electrophoresis in 6% polyacrylamide gel, the DNA-protein complexes were transferred to membrane. The procedure for detection of immobilized nucleic acids was according to the protocol provided by the manufacture.

### 2.9 Pathogen infection

The *Magnaporthe oryzae* PO6-6 strain was cultured on an agar medium containing 8% V8 juice (Cambell’s Soup Company, USA) for 2 weeks under fluorescent light. The resulting spores were collected and diluted in distilled water to obtain the concentration of 5 × 10^6^ mL^−1^ for spot inoculation. A solution containing 2 × 10^5^ spores per mL was sprayed onto the fully expanded healthy leaves of 6-week-old plants. Leaf samples were harvested and photographed to calculate the size of the lesions developed in the susceptible plants (Vo et al., 2019).

## 3 Results

### 3.1 Identification of sucrose-induced genes

To identify genes that preferentially responded to exogenously provided sucrose, RNA-seq analysis was performed using Oc cell lines that had been maintained in suspension culture for a long period (Baba et al., 1986). Because the cells were grown in a culture medium containing sucrose, the carbon source was replaced with glucose to deplete sucrose when the cells were sub-cultured. Ten days after subculture, sucrose was added to a culture flask to obtain a final concentration of 3%. As a control, glucose was added to another culture flask to reach the same final concentration. Four hours after adding the sugars, cells were harvested and mRNAs were isolated to construct cDNA libraries. RNA-seq analyses of the libraries showed that the transcript levels of 1,336 genes were at least doubled by the sucrose addition during the 4-h culture period (**Supplementary Figure 1A**). Among these genes, 871 were also induced by glucose, leaving 465 that were sucrose preferential. The analyses also revealed that 1,148 genes were preferentially suppressed by sucrose (**Supplementary Figure 1B**). To validate the RNA-seq data, 12 upregulated and 12 downregulated genes were selected for qRT-PCR analysis, which confirmed that eight and 10 genes belonging to the two groups, respectively, were sucrose responsive (**Supplementary Table 2**). GO analysis of the 465 genes preferentially induced by sucrose showed that the biological terms dominated by sucrose were protein amino acid phosphorylation, defense response, and transport (**Supplementary Figure 1C**).

### 3.2 Sucrose preferentially induces the transcription initiation of *OsWRKY7*

**Figure 1:**
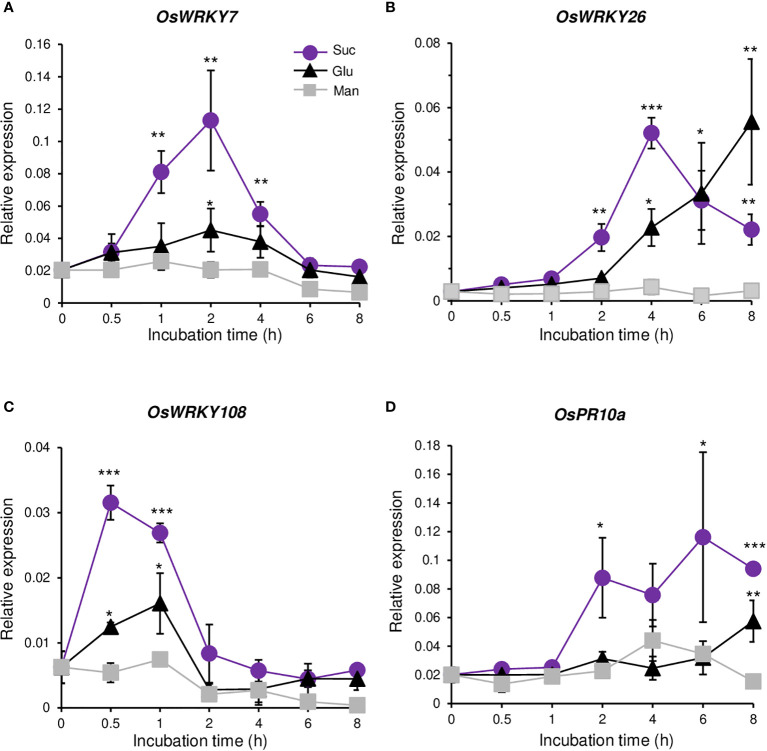
Response to sugars. Quantitative RT-PCR analyses of *OsWRKY7* **(A)**, *OsWRKY26* **(B)**, *OsWRKY108* **(C)**, and *OsPR10a* **(D)** in Oc cells after addition of 3% sucrose (Suc), 3% glucose (Glu) or 3% mannitol (Man). The gene expression levels are relative to those of *OsActin1*. Error bars indicate standard deviations; n = 3 or more. Statistical significance is indicated by *(P < 0.05) **(P < 0.01) and ***(P < 0.001).

Mapman analysis of all the sucrose up-regulated genes from the RNA-seq analysis showed that three groups of transcription factors were enriched in biotic stress (**Supplementary Figure 2**). Because WRKY transcription factors are involved in defense response against pathogens, we selected all three WRKY genes (*OsWRKY7*, *OsWRKY26*, and *OsWRKY108*) that were sucrose inducible. qRT-PCR analysis showed that the transcript level of *OsWRKY7* was increased by sucrose, reaching the maximum level at 2 hours after sucrose addition, and then rapidly declining to the basal level at 6 hours (**Figure 1A**). Glucose also induced the transcript level, but not significantly. Mannitol did not increase gene expression during the 8-h incubation time, which indicated that the expression of *OsWRKY7* was preferentially induced by sucrose. The level of *OsWRKY26* transcript started to increase at 2 hours after sucrose addition, reached the maximum at 4 hours, and then declined (**Figure 1B**). The transcript level was also increased by glucose addition. However, the gene was not induced at 2 hours after glucose addition, but its transcript was increased to approximately half of the sucrose-induced level at 4 hours and continuously increased at 6 and 8 h. The transcript level was not changed by mannitol. The above observations suggest that *OsWRKY26* responded to both sucrose and glucose, but the gene was more rapidly induced by sucrose. Another WRKY gene, *OsWRKY108*, was also induced by sucrose. Compared to the other two genes discussed above, *OsWRKY108* responded more rapidly to sucrose, reaching the maximum level at 0.5 hours after sucrose addition, and declining to the basal level at 2 hours (**Figure 1C**). This gene was weakly induced by glucose and did not respond to mannitol. The pathogen marker gene *OsPR10a* was also preferentially induced by sucrose, but not by glucose or mannitol (**Figure 1D**).

**Figure 2:**
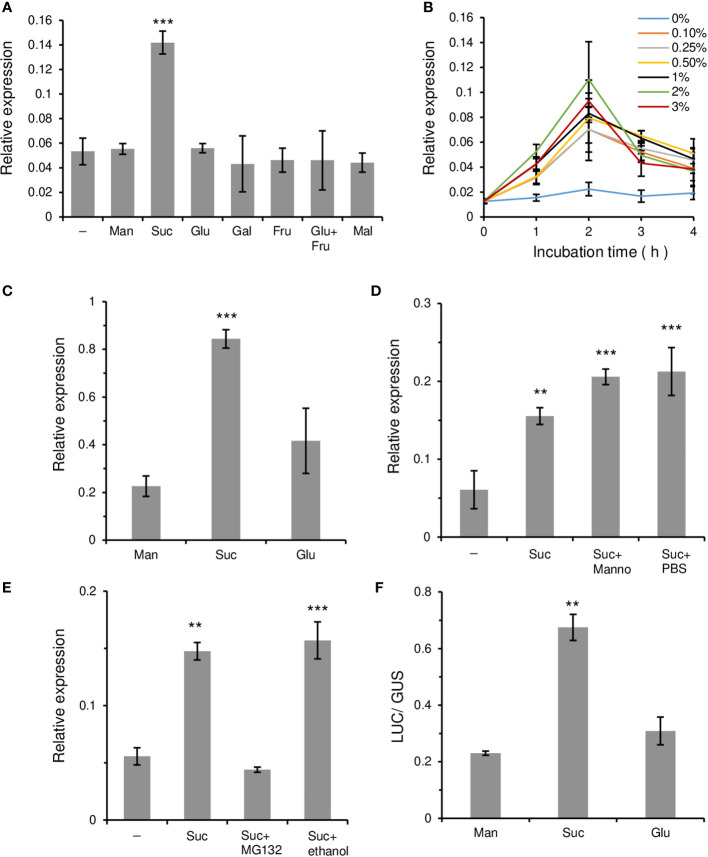
Relative transcripts level of *OsWRKY7* affected by various sugars and inhibitors. **(A-D)** Effects of various sugars and chemicals. **(A)** Effect of sugars at 3% concentration in Oc cells. Samples were collected at 2 hours after sugar addition. **(B)** Effect of different sucrose concentrations. **(C)** Effect of 3% sugars in roots of 14-d-old seedlings. **(D)** Effects of 5 µM mannoheptulose (Manno). Effect of PBS was tested because mannoheptulose was dissolved in PBS before adding to the sucrose-containing medium. **(E)** Influence of MG132. Samples were collected at 1 hours after addition of 3% sucrose, 3% sucrose plus 100 µM MG132, or 3% sucrose plus 0.5% ethanol. We tested the effect of ethanol because MG132 was dissolved in ethanol. **(F)** Luciferase activity driven by the *OsWRKY7* promoter and LUC coding region. *ZmUbi::GUS* construct was used as an internal control. Man, mannitol; Suc, sucrose; Glu, glucose; Gal; galactose; Fru, fructose; Mal, maltose. Minus sign (-) means basal media. Gene expression levels are relative to those of *OsActin1.* The error bars indicate standard deviations; n = 3 or more. Statistical significance is indicated by **(P < 0.01) and ***(P < 0.001).

Because *OsWRKY7* was specifically and transiently induced by sucrose, further tests were conducted to determine whether this gene was induced by other sugars. In addition to glucose, other monosaccharides such as galactose and fructose, as well as glucose and fructose added at the same time, did not induce gene expression above the control level during the 2-h incubation period (**Figure 2A**). Also, gene expression was not stimulated by maltose, a disaccharide that consists of two glucose molecules. The results suggest that *OsWRKY7* is specifically induced by sucrose. Lowering sucrose concentration to 2%, 1%, and 0.5% did not influence the rate and level of *OsWRKY7* induction (**Figure 2B**), however, further reductions to concentrations of 0.25% and 0.1% slightly reduced the sucrose inducibility.

To investigate whether gene expression was induced by sucrose in plants, 14-day-old rice seedlings grown hydroponically in Yoshida solution were left in the dark for 48 hours to reduce their endogenous sugar level. After this period, sucrose was added to the culture medium at the final concentration of 3%. Analysis of the *OsWRKY7* transcript level in the roots showed that it increased significantly at 8 hours after sucrose addition (**Figure 2C**). Glucose also induced gene expression in the roots, but the induced levels were only slightly higher than those achieved by mannitol in the control.

The effect of mannoheptulose, which blocks hexose-mediated signaling (Chiou and Bush, 1998), was also tested. The results showed that the addition of 5 µM mannoheptulose to the sucrose solution did not interfere with the sucrose-induced expression of *OsWRKY7* (**Figure 2D**). This observation suggests that hexoses produced from sucrose metabolism are probably not involved in the induction of the gene and that sucrose may function directly to promote *OsWRKY7* expression.

To elucidate whether protein stability was involved in the sucrose response, MG132, which is an inhibitor of the 26S proteasomal protease (Genschik et al., 1998), was added to the sucrose solution. Analysis of the *OsWRKY7* transcript level showed that the sucrose-induced expression of the gene was suppressed by 100 µM MG132 (**Figure 2E**). As a control, the sucrose-induced *OsWRKY108* gene was also tested and it was shown that the expression of this gene was not affected by MG132 (**Supplementary Figure 3**). This result suggests that a regulatory protein that represses *OsWRKY7* expression is degraded by an E3 ligase during the sucrose treatment.

To examine whether the induction of *OsWRKY7* by sucrose occurred at the transcriptional level, the 3.081-kb promoter sequence of *OsWRKY7* was placed upstream of the *LUC* coding region and the construct was introduced into protoplasts prepared from Oc cells. As an internal control, the *GUS* coding region driven by the *ZmUbi* promoter was co-transfected. After dividing the treated cells into three portions, sucrose, glucose, and mannitol at the final concentration of 3% were added to each aliquot. After 15 hours of incubation, the samples were analyzed and it was shown that the LUC activity in the sucrose-added cells was considerably higher than that in the mannitol-added cells (**Figure 2F**). On the contrary, the induction of the reporter gene expression by glucose was only slightly above that achieved by mannitol. This experiment demonstrated that sucrose preferentially induced *OsWRKY7* at the transcription initiation level.

### 3.3 Mutations of *OsWRKY7* reduce the defense response against *M. oryzae*

Expression of *OsWRKY7* was induced by *M. oryzae* 48 hours after infection (**Supplementary Figure 4A**). To study the functional roles of *OsWRKY7*, we identified T-DNA tagging line 5A-00022, in which T-DNA was inserted at 375 bp upstream of the start ATG codon of *OsWRKY7* (**Supplementary Figure 4B**). Homozygous progeny of the *oswrky7-1* mutant was used for characterization of the gene function. In the mutant line, the expression level of *OsWRKY7* was decreased to approximately a half of the wild type (WT) level, which indicated that the inserted T-DNA reduced gene expression (**Supplementary Figure 4C**). When the plants were infected with *M. oryzae*, the lesions were longer and broader than those observed in the segregated WT plants (**Supplementary Figures 4D, E**), suggesting that *OsWRKY7* is involved in the defense response against this pathogen.

**Figure 3:**
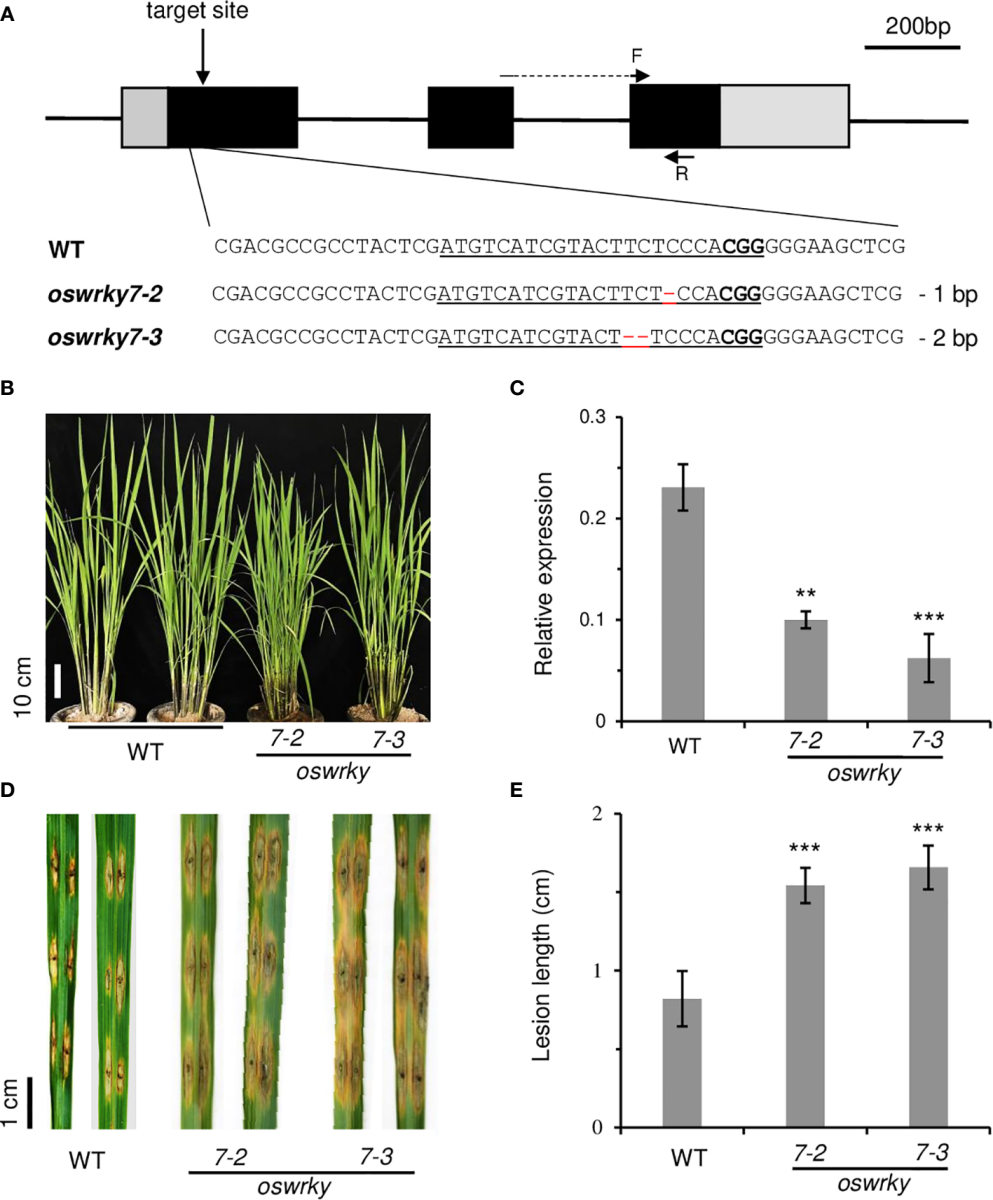
Characterization of *oswrky7* mutants generated by the CRISPR/Cas9 method. **(A)** Schematic diagram of *OsWRKY7* gene structure and sequence comparison of sgRNA target regions between WT and mutants. The target sequence and PAM site are underlined. Deleted sequences are indicated with red dashes. In the schematic gene structure, black boxes are exons; gray boxes are 5’- UTR and 3’-UTR regions; lines between the boxes are introns. F and R are primers used for measuring the transcript level of *OsWRKY7*. Scale bar = 200 bp. **(B)** Phenotypes of the WT and the knockout mutant plants grown under the paddy field at T1 generation. Scale bar = 10 cm. **(C)** Transcript level of *OsWRKY7* in the WT and knockout mutant plants. Samples were collected at 42 DAG **(D)** Comparison of the lesions between the WT and mutants. Phenotype was observed at 9 days after *M. oryzae* infection. Scale bar = 1 cm. **(E)** The length of the lesion developed by the pathogen. The error bars show standard deviations; n = 10. Statistical significance is indicated by **(P < 0.01) and ***(P < 0.001).

To confirm this observation, knockout mutations were generated in the first exon of *OsWRKY7 via* the CRISPR/Cas9 system (**Figure 3A**). Among 28 independent transgenic plants, five were selected to determine the flanking regions of the target site. Two mutant lines were chosen: *oswrky7-2* carrying a single-bp deletion and *oswrky7-3* carrying a two-bp deletion (**Figure 3A**, **Supplementary Figures 5A, B**). The deletions generated frameshift mutations, causing early termination during translation of the *OsWRKY7* transcript. The mutant plants grew normally without any significant phenotypic alteration in the plant height, grain numbers per panicle, panicle length, and 100-grain weight (**Figure 3B**, **Supplementary Figure 6**). Interestingly, the *OsWRKY7* transcript levels in the frameshift mutants were reduced compared to those in the WT plants, suggesting that the deletion mutations affected either the transcription rate or the stability of the transcript (**Figure 3C**). When the plants were inoculated with *M. oryzae*, the mutants displayed a phenotype that was more susceptible to the fungus compared with the WT controls (**Figures 3D, E**). These experiments confirmed that *OsWRKY7* is involved in the defense response against *M. oryzae*.

### 3.4 Overexpression of *OsWRKY7* increases the defense response against *M. oryzae*

**Figure 4:**
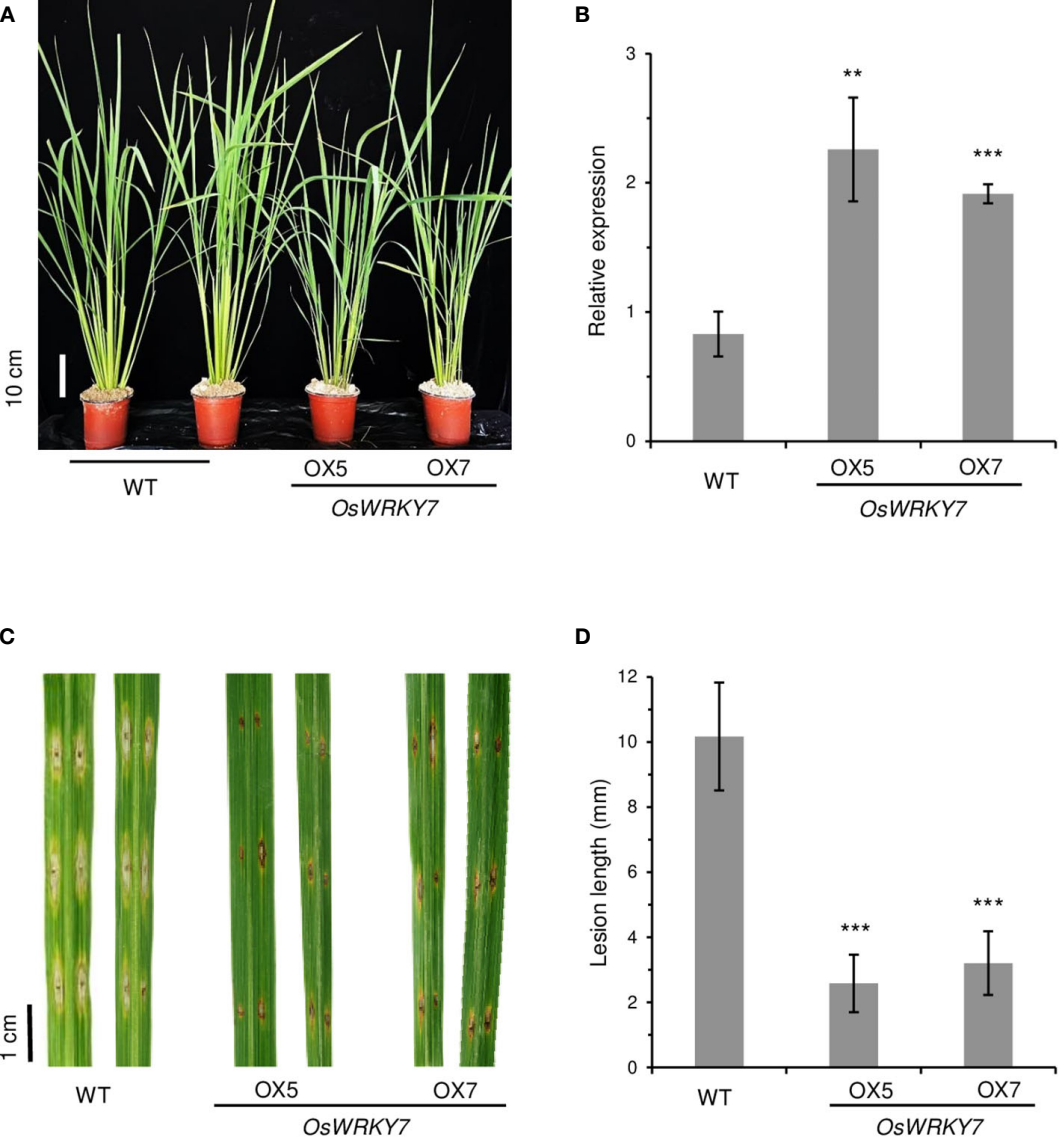
Phenotypes of *OsWRKY7* overexpression plants. **(A)** Phenotype of the WT and transgenic plants overexpressing *OsWRKY7.* Scale bar = 10 cm. **(B)** The relative transcript level of *OsWRKY7* in the WT and transgenic plants OX5 and OX7 overexpressing *OsWRKY7*. The gene expression levels are relative to those of *OsActin1*. Errors bars indicate standard deviation; n = 3. **(C)** Phenotypes of the lesions at 9 days after infection with *M. oryzae*. Bar = 1 cm. **(D)** The length of the lesion developed by the pathogen. Errors bars indicate standard deviation; n = 10. Statistical significance is indicated by **(P < 0.01) and ***(P < 0.001).

To obtain transgenic rice plants that constitutively expressed *OsWRKY7*, the gene was placed after the *ZmUbi* promoter and the construct was introduced to rice embryonic calli. However, it was quite difficult to obtain transgenic plants, which suggested that the overexpression of *OsWRKY7* was harmful to the plants. To overcome the difficulty, the HA tag was added at the end of the *OsWRKY7* coding region, and the fusion molecule was placed after the *ZmUbi* promoter. In this way, several transgenic plants expressing *OsWRKY7*-*HA* were obtained, which indicated that attaching the tag reduced the harmful effect of the gene (**Figure 4A**). Plants #5 and #7, which expressed the fusion transcript and the HA-tagged protein at high levels, were selected (**Figure 4B** and **Supplementary Figure 7**). After inoculating the mature leaves of the transgenic plants with *M. oryzae*, it was observed that the lesion lengths and widths at the infection sites were significantly smaller in the transgenic plants than in the WT control plants (**Figures 4C, D**). This observation confirmed that *OsWRKY7* is a positive regulatory element in the defense response to *M. oryzae*.

**Figure 5:**
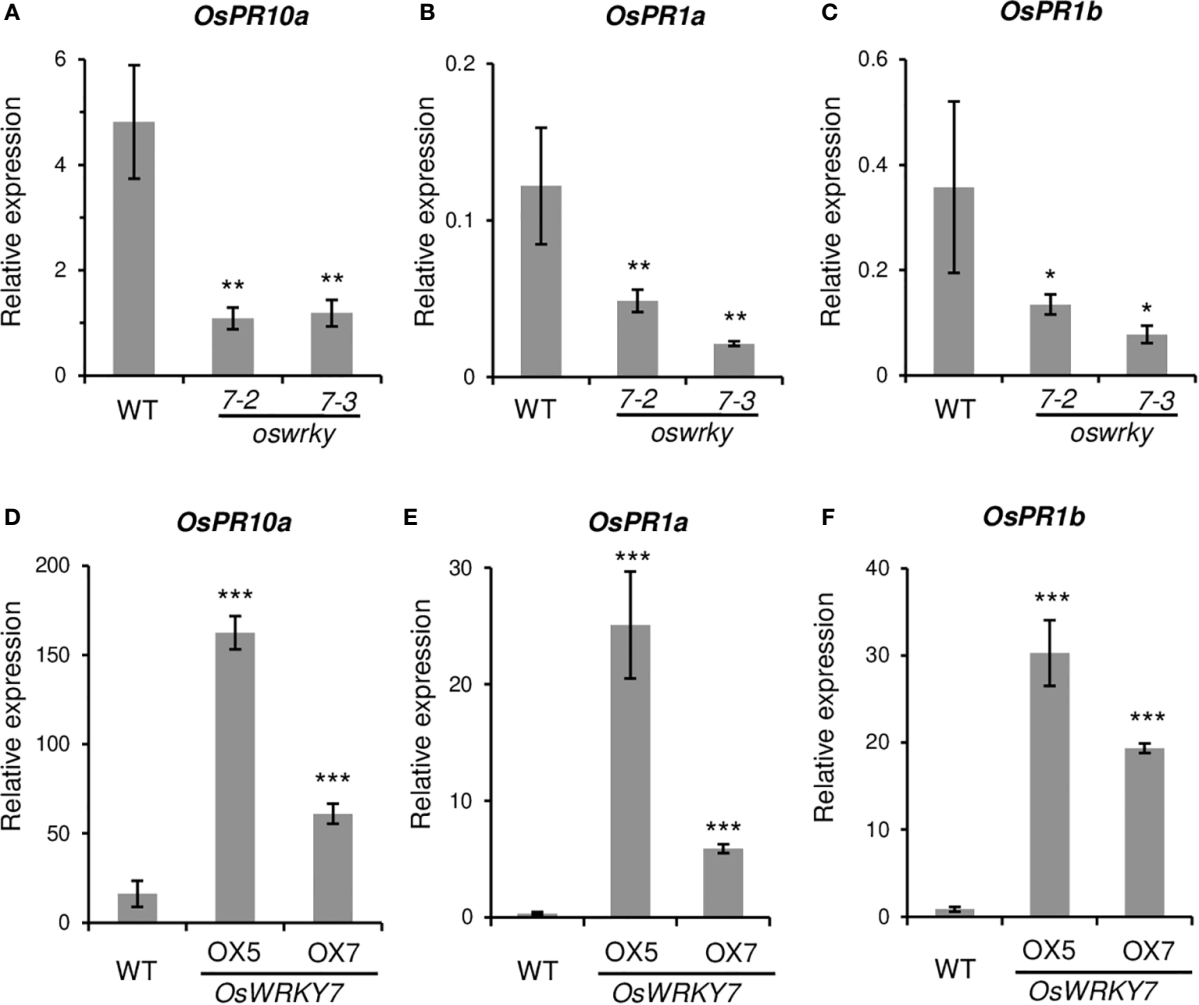
Expression levels of pathogen related genes in the WT and transgenic plants. **(A-C)** Relative transcript levels of *OsPR10a* **(A)**, *OsPR1a* **(B)** and *OsPR1b* **(C)** in the leaves of the WT and *oswrky7* mutants. **(D-F)** Relative transcript levels of *OsPR10a* **(D)**, *OsPR1a* **(E)** and *OsPR1b* **(F)** in the leaves of the WT and *OsWRKY7* overexpression plants. Samples were collected from the leaf blades at 42 DAG. The gene expression levels are relative to those of *OsActin1*. Error bars indicate standard deviation; n = 3 or more. Statistical significance is indicated by *(P < 0.05), **(P < 0.01) and ***(P < 0.001).

The expression level of the genes that were induced in response to pathogen infection were then analyzed. The levels of the pathogen-responsive marker genes *OsPR1a*, *OsPR1b*, and *OsPR10a*, were significantly reduced in *oswrky7* mutant plants (**Figures 5A-C**). On the contrary, expression levels of the pathogen responsive marker genes were markedly increased in the plants of expressing *OsWRKY7*-*HA* (**Figures 5D-F**). This result indicates that the markers function downstream of *OsWRKY7*. It was then tested whether SA or JA were involved in the induction of the marker genes by *OsWRKY7*. The mutations of this gene did not affect the expression of *phenylalanine ammonia-lyase* (*OsPAL*) or *isochorismate synthase 1* (*OsICS1*), which are involved in SA biosynthesis (**Supplementary Figures 8A, B**). In addition, a key regulator of systemic acquired resistance, *NPR1 homolog 1* (*OsNH1*), was also not affected in mutant (**Supplementary Figure 8C**). Likewise, the mutations did not influence the expression of genes encoding enzymes associated with JA biosynthesis, namely *allene oxide synthase* (*OsAOS2*), *allene oxide cyclase 1* (*OsAOC1*), and *oxophytodienoate reductase 3* (*OsOPR3*) (**Supplementary Figures 8D-F**). These results suggest that the signaling pathway of SA or JA did not mediate the induction of the pathogen-responsive marker genes by *OsWRKY7*.

**Figure 6:**
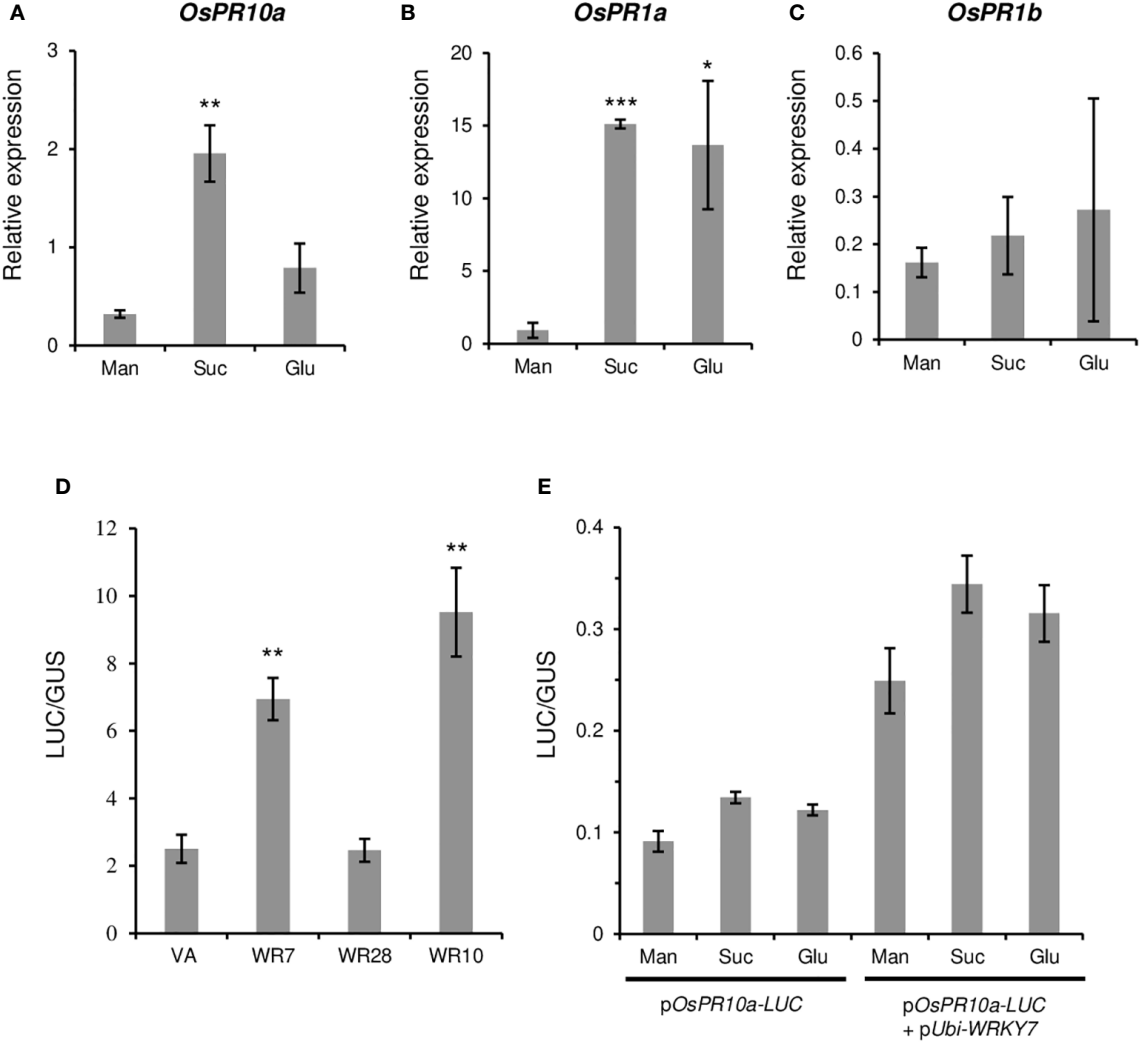
Effect of sugars and WRKY genes on the PR gene expression. **(A-C)** Effect of sugars on PR gene expression. Fourteen-day-old seedlings were grown in a hydroponic culture medium under constant light and they were transferred to the dark condition for two additional days before sucrose treatment. Mannitol, sucrose, or glucose were added at 3% final concentration. After the treatment for 8 hours, expression levels of *OsPR10a* **(A)**, *OsPR1a* **(B)**, and *OsPR1b* **(C)** in roots were measured. Gene expression levels are relative to those of *OsActin1*. Error bars indicate standard deviations; n = 3 or more. **(D)** Luciferase activity driven by the *OsPR10a* promoter. The p*OsPR10a-LUC* construct was transfected to Oc cell protoplasts alone (VA) or with p*Ubi-OsWRKY7* (WR7), p*Ubi-OsWRKY10* (WR10), or p*Ubi-OsWRKY28* (WR28). The LUC activity was assayed 15 hours after transfection of the molecules. *ZmUbi-GUS* construct was used as an internal control in each combination. **(E)** Effect of sugars on the *OsPR10a* promoter activity. p*OsPR10a-LUC* alone or together with p*Ubi-WRKY7* were transferred to Oc protoplasts and incubated in a culture medium containing mannitol (Man), sucrose (Suc) or glucose (Glu) at 3% final concentration. Luciferase activity was measured 15 hours after transfection. *ZmUbi-GUS* construct was used as an internal control in each experiment. Error bars indicate standard deviations; n = 3. Statistical significance is indicated by *(P < 0.05), **(P < 0.01) and ***(P < 0.001).

To evaluate whether the *OsWRKY7*-induced PR genes were sucrose-inducible, hydroponically grown seedlings were treated with 3% sucrose at 14 days after germination (DAG). After 8 hours of incubation in the solution, the expression levels of the PR genes in the roots were measured. The assay showed that the transcript level of *OsPR10a* was considerably higher in the sucrose-treated seedlings than in the control seedlings treated with 3% mannitol (**Figure 6A**). Glucose treatment did not induce gene expression above the level observed in the control. This result is consistent with the data observed in Oc cells (**Figure 2D**). On the contrary, the expression of *OsPR1a* was induced by both sucrose and glucose (**Figure 6B**). The *OsPR1b* transcript levels in sugar solutions were similar to those observed in mannitol solution (**Figure 6C**). These experiments indicate that, among the three PR genes tested, only *OsPR10a* was preferentially induced by sucrose.

We tested whether *OsPR10a* expression in *oswrky7* mutant was recovered by the sucrose treatment. The WT and *oswrky7* plants that were grown hydroponically in Yoshida solution for 14 days were treated with mannitol or sucrose at the final concentration of 3%. After 9 hours treatment in the solutions, the root were sampled and the expression level of *OsPR10a* was measured. The experiment showed that sucrose significantly increased *OsPR10a* expression in the WT as observed in the previous experiments. Similarly, the transcript level of the gene in the mutant was also induced by sucrose, although the induced level was lower compared with WT (**Supplementary Figure 9**). This observation suggests that there are other sucrose-inducible transcription factors that induce *OsPR10a* expression.

### 3.5 Expression of *OsPR10a* is stimulated by *OsWRKY7*

Because the expression of *PR10a* was significantly reduced in *OsWRKY7* mutants, *PR10a* may be directly controlled by the transcription factor. To prove this hypothesis, a construct containing the 2.856-kb promoter region of *OsPR10a* was placed in front of the *LUC* gene. We also constructed another molecule in which the coding region of *OsWRKY7* was placed under the *ZmUbi* promoter. Both molecules, p*OsPR10a-LUC* and p*Ubi*-*WRKY7*, were co-transfected into the Oc cell protoplasts and incubated for 15 hours to express the introduced genes. The results showed that the *LUC* expression level in the protoplasts co-transferred with both molecules were significantly higher than that in the protoplasts transferred with p*OsPR10a-LUC* alone (**Figure 6D**). As controls, *OsWRKY10* and *OsWRKY28* were inserted downstream of the *ZmUbi* promoter. It has been previously reported that *OsWRKY10* functions as a positive regulatory element for the expression of *PR10a*, whereas *OsWRKY28* does not influence the PR gene (Ersong et al., 2021). In this study, when p*Ubi*-*WRKY28* was co-introduced with p*OsPR10a-LUC*, the luciferase activity was not increased above the background level that was observed from p*OsPR10a-LUC* alone (**Figure 6D**). On the contrary, co-transfection of p*OsPR10a-LUC* and p*Ubi*-*OsWRKY10* significantly induced the reporter gene expression (**Figure 6D**). The above observations showed that the transient assay resembled the previous results and suggested that OsWRKY7 directly promoted the expression of p*OsPR10a-LUC*.

To test whether the *OsPR10a* promoter region responded directly to sucrose, the p*OsPR10a-LUC* vector was introduced into the protoplasts and was incubated in the culture solution containing three different sugars. Although the *LUC* expression level in the protoplasts cultured in sucrose-containing medium was slightly higher than that in the mannitol control, a similar expression level was achieved for the protoplasts grown in the glucose-containing medium (**Figure 6E**). This suggests that sucrose did not induce the gene directly. It was also tested whether the increased expression of the *OsPR10a* promoter by the OsWRKY7 protein was sucrose dependent. To answer this question, both p*OsPR10a-LUC* and p*Ubi*-*WRKY7* were introduced into the protoplasts and the effects of sucrose were examined. The experiment showed that sucrose did not significantly increase the p*OsPR10a* promoter activity above the levels obtained from mannitol or glucose (**Figure 6E**). This suggests that the sucrose induction of *PR10a* was not due to the stability of the OsWRKY7 mRNA or protein during sucrose treatments.

**Figure 7:**
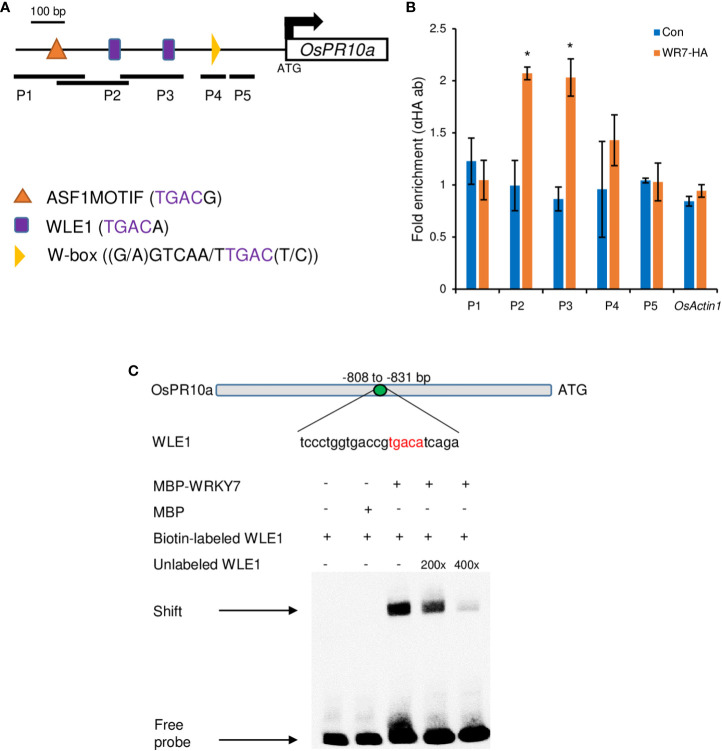
ChIP assay on the OsPR10a chromatin region and EMSA experiment. **(A)** Promoter region of *OsPR10a*. The black lines under the promoter region indicate the DNA fragment amplified by RT-PCR. Five pairs of primer set were used in ChIP assay; P1, 849-967 bp from the ATG start codon; P2, 795-946 bp from the ATG start codon; P3, 647-801 bp from the ATG start codon; P4, 207-289 bp from the ATG start codon; P5, 102-205 bp from the ATG start codon. Scale bar = 100 bp. **(B)** Chromatin enrichment of the *OsPR10a* promoter region. Transgenic plants expressing *OsWRKY7-HA* were used for the ChIP assay. Error bars indicate standard deviations; n = 2. Statistical significance is indicated by *(P < 0.05). **(C)** EMSA experiment using the sequence that contains WLE1 element on the promoter of *OsPR10a*. Biotin-labeled probe WLE1 without MBP-OsWRKY7 protein and biotin-labeled WLE1 with MBP protein were used as negative control. Excess unlabeled WLE1 was used as competitors.

To confirm that OsWRKY7 directly controlled the *OsPR10a* promoter, chromatin immunoprecipitation (ChIP) experiments were performed with transgenic plants expressing the OsWRKY7-HA protein. Primers were designed to target W-box elements, W-box like elements 1 (WLE1), and ASF1MOTIF, which all belong to the WRKY protein-binding TGAC core elements (Maleck et al., 2001; Hwang et al., 2008) (**Figure 7A**). In the ChIP *in vivo* assay, the P2 and P3 DNA fragments containing the WLE1 element were enriched (**Figure 7B**).

To further confirm the interaction between OsWRKY7 and the OsPR10a promoter, the oligonucleotide (-808 to - 831 from the start ATG) containing the WLE1 element within the P2 fragment were used for EMSA experiment using MBP tagged OsWRKY7 protein (**Figure 7C**). The experiment showed that OsWRKY7 protein bind to the sequence and the binding was reduced when the unlabeled probe was used as a competitor. Both ChIP and EMSA assays support the hypothesis that OsWRKY7 directly binds to the promoter region of *OsPR10a.*

## 4 Discussion

A hypothesized model for high sugar resistance suggested that a yet unknown sugar receptor senses the elevation of the levels of extracellular sugars and triggers defense through a signaling cascade (Bezrutczyk et al., 2018). Notably, it was shown that rice plants treated with sucrose through the root system exhibited an accumulation of transcripts associated with defense genes in leaves and increased resistance to *M. oryzae* (Gómez-Ariza et al., 2007). This is consistent with previous results indicating that transgenic tobacco and rice plants expressing *PRms* accumulated sucrose in leaf tissues and enhanced their defense response (Murillo et al., 2003; Gómez-Ariza et al., 2007). As PRms is localized in plasmodesmata in infected maize radicles (Murillo et al., 1997), the accumulation of sucrose is possibly due to an alteration of sugar distribution. These observations clearly suggest the implication of plants’ sucrose dependent responses in the defense against invading pathogens. However, it remains to be determined how high levels of sucrose can regulate defense genes in plants.

In the present study, to investigate the functions of sucrose in the defense response, we examined *OsWRKY7*, which is inducible by exogenously applied sucrose. The transcript level of this gene was rapidly increased by the addition of sucrose in both cultured cells and seedlings, suggesting that this sugar functions as a signal molecule to induce gene expression.

Although sucrose controls various processes, such as starch metabolism, storage proteins, and anthocyanin biosynthesis, studies on sucrose responsive transcription factors that may be involved in sucrose signaling are scarce (Yoon et al., 2021). In *Arabidopsis*, sucrose-induced anthocyanin biosynthesis is mediated by *Myb75* (Teng et al., 2005); however, other sugars, such as glucose and fructose, also stimulate anthocyanin accumulation. In addition, mannoheptulose, which is a competitive inhibitor of hexokinase1, inhibits sucrose induction (Neta-Sharir et al., 2000). Therefore, *Myb75* may not be a mediator of sucrose-specific signaling. Another transcription factor that is induced by sucrose is *AtWRKY20* (Nagata et al., 2012). This regulatory gene controls the expression of *ApL3*, which encodes AGPase in *Arabidopsis*. Similarly, the barley starch synthesis gene *isoamylase1* is regulated by the sucrose-inducible WRKY gene *SUSIBA2* (Sun et al., 2003). However, it has not been tested whether these WRKY genes are specifically induced by sucrose. In this study, we demonstrated that sucrose preferentially controlled the expression of *OsWRKY7* because hexoses and mannose were unable to induce this gene. Mannoheptulose did not reduce the sucrose induction of the gene, suggesting that glucose or other hexoses metabolized from sucrose were not responsible for this process.

Sucrose regulates gene expression at the transcriptional or post-transcriptional level. It increases the transcript levels of the WRKY and *Myb75* genes, but controls the translation of the *bZIP11* transcript post-transcriptionally (Wiese et al., 2004). In the present study, the expression of the *LUC* reporter gene driven by the 3-kb *OsWRKY7* promoter sequence was shown to be increased by sucrose, which indicated that sucrose regulates *OsWRKY7* at the transcriptional level.

Because several WRKY genes are involved in plant defense mechanisms, we tested whether *OsWRKY7* functions in disease tolerance against *M. oryzae* using a knockdown mutant generated by T-DNA tagging and knockout mutants created by CRISPR/Cas9. These mutants were more susceptible to the rice blast fungus, however, transgenic plants overexpressing the gene showed reduced disease symptoms. These results suggest that the transcription factor functions positively in the defense against *M. oryzae.* The transcript levels of the pathogen-related genes *OsPR1a*, *OsPR1b*, and *OsPR10a* were significantly reduced in the mutants and increased in the plants exhibiting overexpression, supporting the hypothesis that *OsWRKY7* plays a role in plant defense. However, genes involved in SA synthesis or SA-mediated resistance were not affected by the mutations, and this was the case also for JA biosynthesis genes, suggesting that *OsWRKY7* functions independently from the hormones.

Interestingly, the transcript level of *OsWRKY7* was significantly reduced when MG132 was added during sucrose induction, which indicates that an upstream repressor protein of *OsWRKY7* may be degraded through the proteasome pathway and that sucrose promotes this pathway. AtWRKY50, an ortholog of OsWRKY7, interacts with *Botrytis*-induced kinase 1 (BIK1), a receptor-like cytoplasmic kinase VII member that is localized in the nucleus and degraded by the proteasome (Lal et al., 2018). This suggests that OsWRKY7 may interact with a kinase. Further study will be needed to investigate how the protein is degraded by a proteasome.

Among the three PR genes that were influenced by *OsWRKY7*, only *OsPR10a* was preferentially induced by sucrose. *OsPR1a* was also induced by glucose, which indicates that other regulatory elements besides OsWRKY7 also control its expression. In contrast, *OsPR1b* was not induced by either sucrose or glucose. Because *OsPR1b* also functions downstream of the sucrose-inducible *OsWRKY7* gene, the induction of this PR gene by sucrose was expected. It will be interesting to investigate how the sucrose signaling was diminished in the PR gene. To study whether the induction of *PR10a* expression by sucrose occurred directly or was mediated by OsWRKY7 protein, the promoter of *PR10a* was tested using the *LUC* reporter system. It was shown that sucrose did not induce the promoter activity of *OsPR10a*. Ru et al. (2015) reported that GFP fused OsWRKY7 protein is localized in the nucleus and OsWRKY7 protein has transcriptional activity in yeast hybrid assay, which indicated that the sucrose induction of the *PR10a* promoter was mediated by OsWRKY7. Direct control of this PR gene by OsWRKY7 was also confirmed by the ChIP and EMSA assays. All the results presented in this study provide a definitive proof that sucrose has a role in defense response in rice by activating the transcription factor OsWRKY7, which triggers the expression of *PR10a*. Further experiment is needed to investigate what is the upstream element that mediates sucrose signaling and whether OsWRKY7 interacts with a partner to control the downstream gene expression.

## Data availability statement

The datasets presented in this study can be found in online repositories. The names of the repository/repositories and accession number(s) can be found in the article/**Supplementary Material**.

## Author contributions

GA, S-WL, K-HJ, J-SJ designed the project. WT, JY, KV, L-HC, TH, XP, E-JK, and KW performed experiments and analyzed data. WT, JY, J-SJ, and GA wrote the paper. All authors contributed to the article and approved the submitted version.
