# Supplementary material for: Sucrose preferentially promotes expression of OsWRKY7 and OsPR10a to enhance defense response to blast fungus in rice
Source: Front Plant Sci. 2023 Jan 27;14:1117023. doi: 10.3389/fpls.2023.1117023 (PMC9911862; doi:10.3389/fpls.2023.1117023)
Supplement: Supplementary file 1 [file Presentation_1.pptx]

## Slide 1
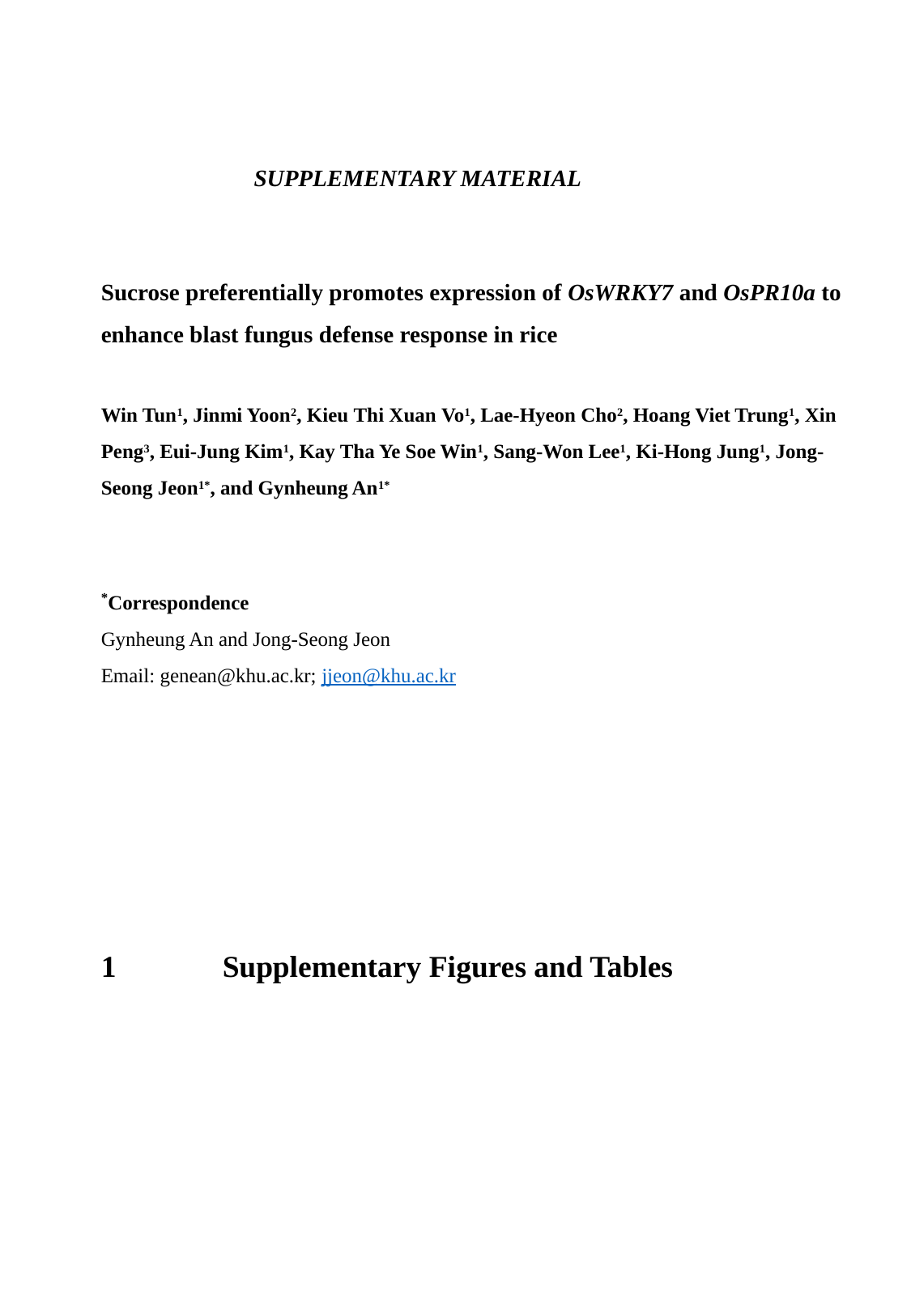

Supplementary Material
Sucrose preferentially promotes expression of OsWRKY7 and OsPR10a to enhance blast fungus defense response in rice
Win Tun1, Jinmi Yoon2, Kieu Thi Xuan Vo1, Lae-Hyeon Cho2, Hoang Viet Trung1, Xin Peng3, Eui-Jung Kim1, Kay Tha Ye Soe Win1, Sang-Won Lee1, Ki-Hong Jung1, Jong-Seong Jeon1*, and Gynheung An1*
*Correspondence
Gynheung An and Jong-Seong Jeon
Email: genean@khu.ac.kr; jjeon@khu.ac.kr
1	Supplementary Figures and Tables

## Slide 2
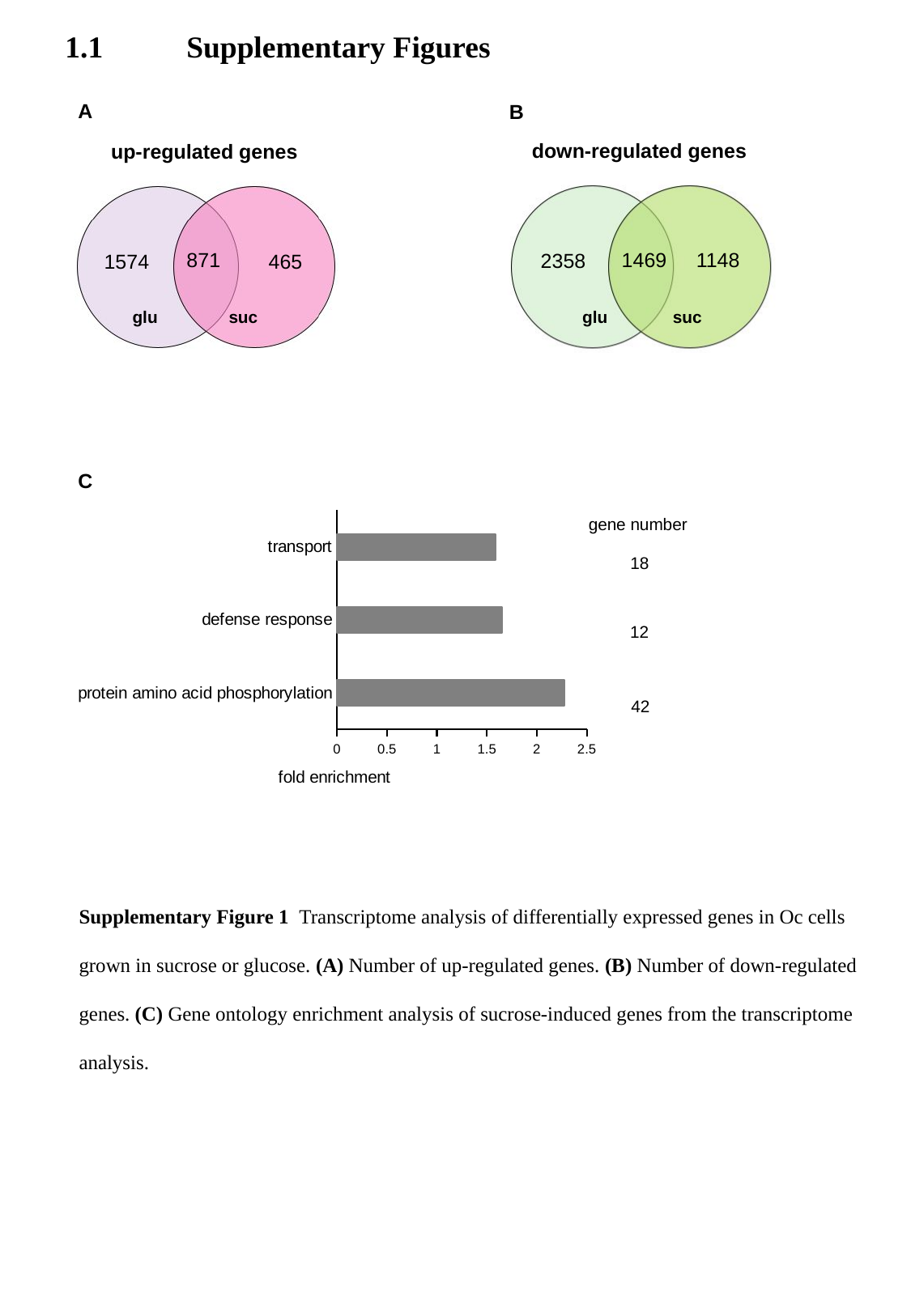

1.1	Supplementary Figures
A
B
 down-regulated genes
suc
glu
 up-regulated genes
suc
glu
1469
1148
871
2358
465
1574
C
### Chart
| Category | |
|---|---|
| protein amino acid phosphorylation | 2.272997867711524 |
| defense response | 1.6526194017517766 |
| transport | 1.5867139154810386 |18
12
42
gene number
Supplementary Figure 1 Transcriptome analysis of differentially expressed genes in Oc cells grown in sucrose or glucose. (A) Number of up-regulated genes. (B) Number of down-regulated genes. (C) Gene ontology enrichment analysis of sucrose-induced genes from the transcriptome analysis.

## Slide 3
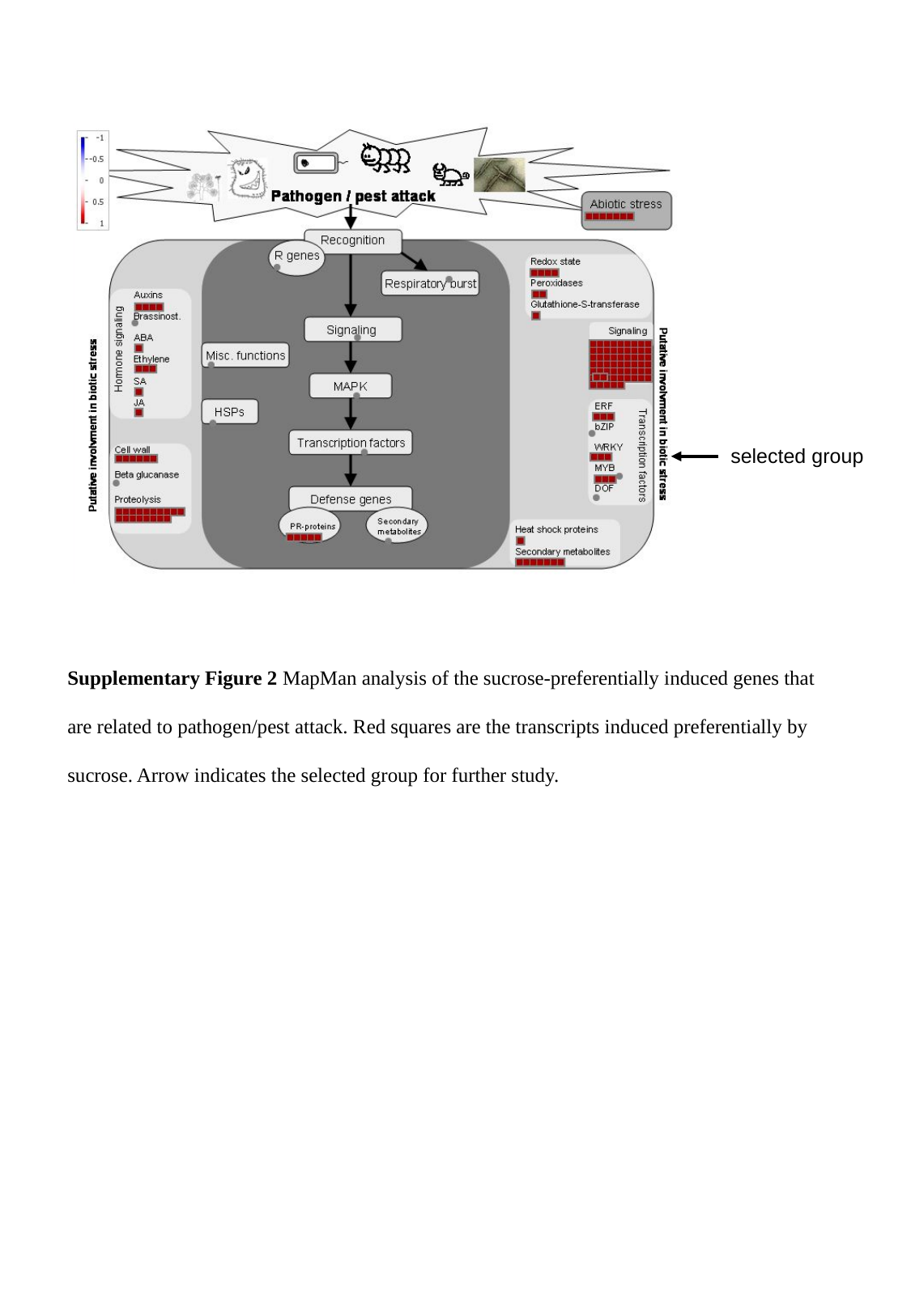

selected group
Supplementary Figure 2 MapMan analysis of the sucrose-preferentially induced genes that are related to pathogen/pest attack. Red squares are the transcripts induced preferentially by sucrose. Arrow indicates the selected group for further study.

## Slide 4
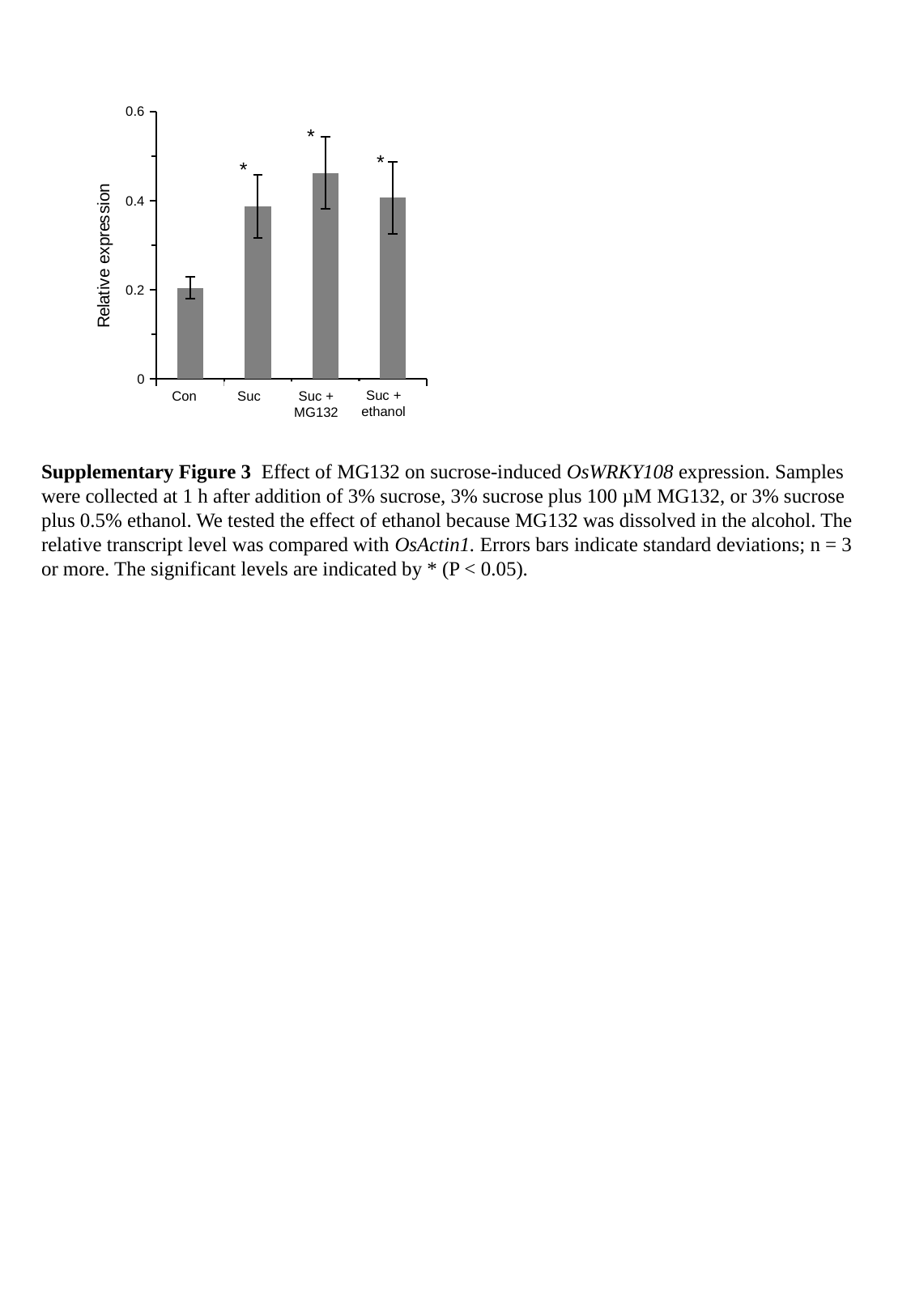

### Chart
| Category | |
|---|---|
| Con | 0.2041148935400965 |
| Suc | 0.387218698001 |
| MG132 | 0.462369473831359 |
| Ethanol | 0.40635756668562645 |*
*
*
Suc +
ethanol
Suc +
MG132
Con
Suc
Supplementary Figure 3 Effect of MG132 on sucrose-induced OsWRKY108 expression. Samples were collected at 1 h after addition of 3% sucrose, 3% sucrose plus 100 µM MG132, or 3% sucrose plus 0.5% ethanol. We tested the effect of ethanol because MG132 was dissolved in the alcohol. The relative transcript level was compared with OsActin1. Errors bars indicate standard deviations; n = 3 or more. The significant levels are indicated by * (P < 0.05).

## Slide 5
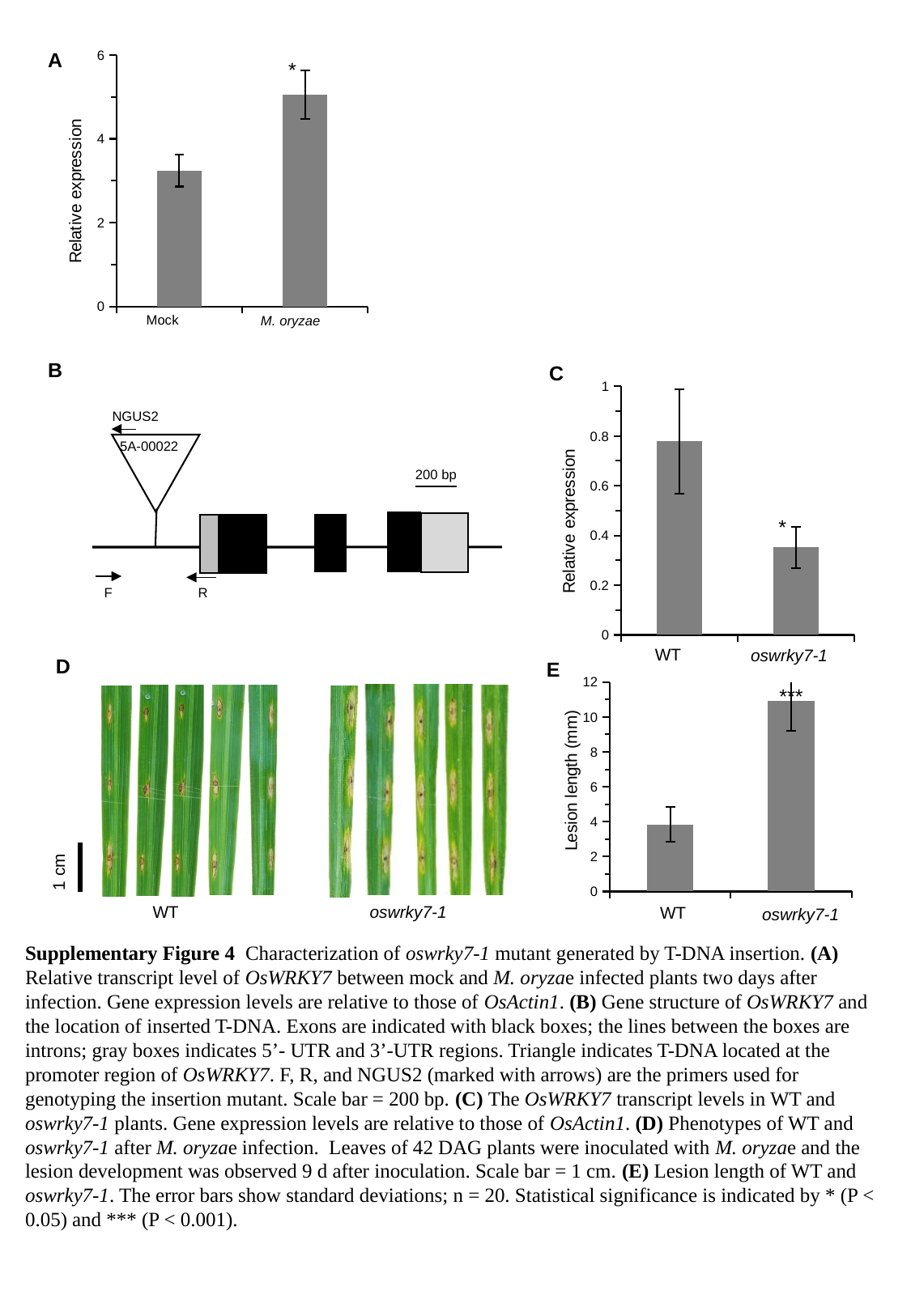

A
### Chart
| Category | |
|---|---|
| Mock | 3.241935197238616 |
| M. oryzae | 5.051767444659363 |*
Mock
M. oryzae
B
C
### Chart
| Category | |
|---|---|
| WT | 0.7788779655283822 |
| mutant | 0.3516373144233468 |*
WT
oswrky7-1
NGUS2
5A-00022
200 bp
F
R
D
E
### Chart
| Category | |
|---|---|
| Wild type | 3.8333333333333335 |
| Mutant | 10.935185185185185 |***
WT
oswrky7-1
oswrky7-1
WT
1 cm
Supplementary Figure 4 Characterization of oswrky7-1 mutant generated by T-DNA insertion. (A) Relative transcript level of OsWRKY7 between mock and M. oryzae infected plants two days after infection. Gene expression levels are relative to those of OsActin1. (B) Gene structure of OsWRKY7 and the location of inserted T-DNA. Exons are indicated with black boxes; the lines between the boxes are introns; gray boxes indicates 5’- UTR and 3’-UTR regions. Triangle indicates T-DNA located at the promoter region of OsWRKY7. F, R, and NGUS2 (marked with arrows) are the primers used for genotyping the insertion mutant. Scale bar = 200 bp. (C) The OsWRKY7 transcript levels in WT and oswrky7-1 plants. Gene expression levels are relative to those of OsActin1. (D) Phenotypes of WT and oswrky7-1 after M. oryzae infection. Leaves of 42 DAG plants were inoculated with M. oryzae and the lesion development was observed 9 d after inoculation. Scale bar = 1 cm. (E) Lesion length of WT and oswrky7-1. The error bars show standard deviations; n = 20. Statistical significance is indicated by * (P < 0.05) and *** (P < 0.001).

## Slide 6
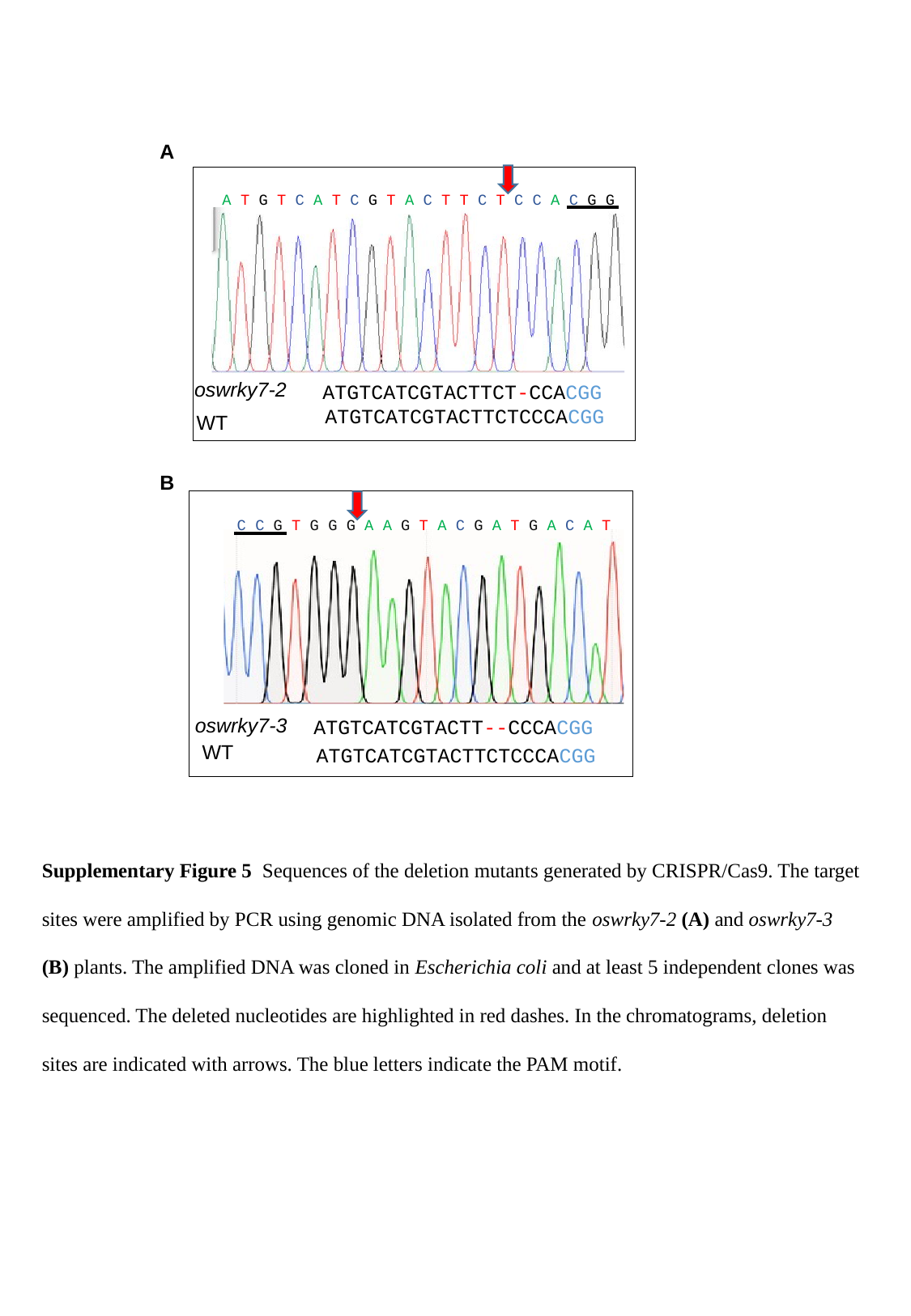

A
A T G T C A T C G T A C T T C T C C A C G G
ATGTCATCGTACTTCT-CCACGG
ATGTCATCGTACTTCTCCCACGG
oswrky7-2
WT
B
C C G T G G G A A G T A C G A T G A C A T
oswrky7-3
ATGTCATCGTACTT--CCCACGG
WT
ATGTCATCGTACTTCTCCCACGG
Supplementary Figure 5 Sequences of the deletion mutants generated by CRISPR/Cas9. The target sites were amplified by PCR using genomic DNA isolated from the oswrky7-2 (A) and oswrky7-3 (B) plants. The amplified DNA was cloned in Escherichia coli and at least 5 independent clones was sequenced. The deleted nucleotides are highlighted in red dashes. In the chromatograms, deletion sites are indicated with arrows. The blue letters indicate the PAM motif.

## Slide 7
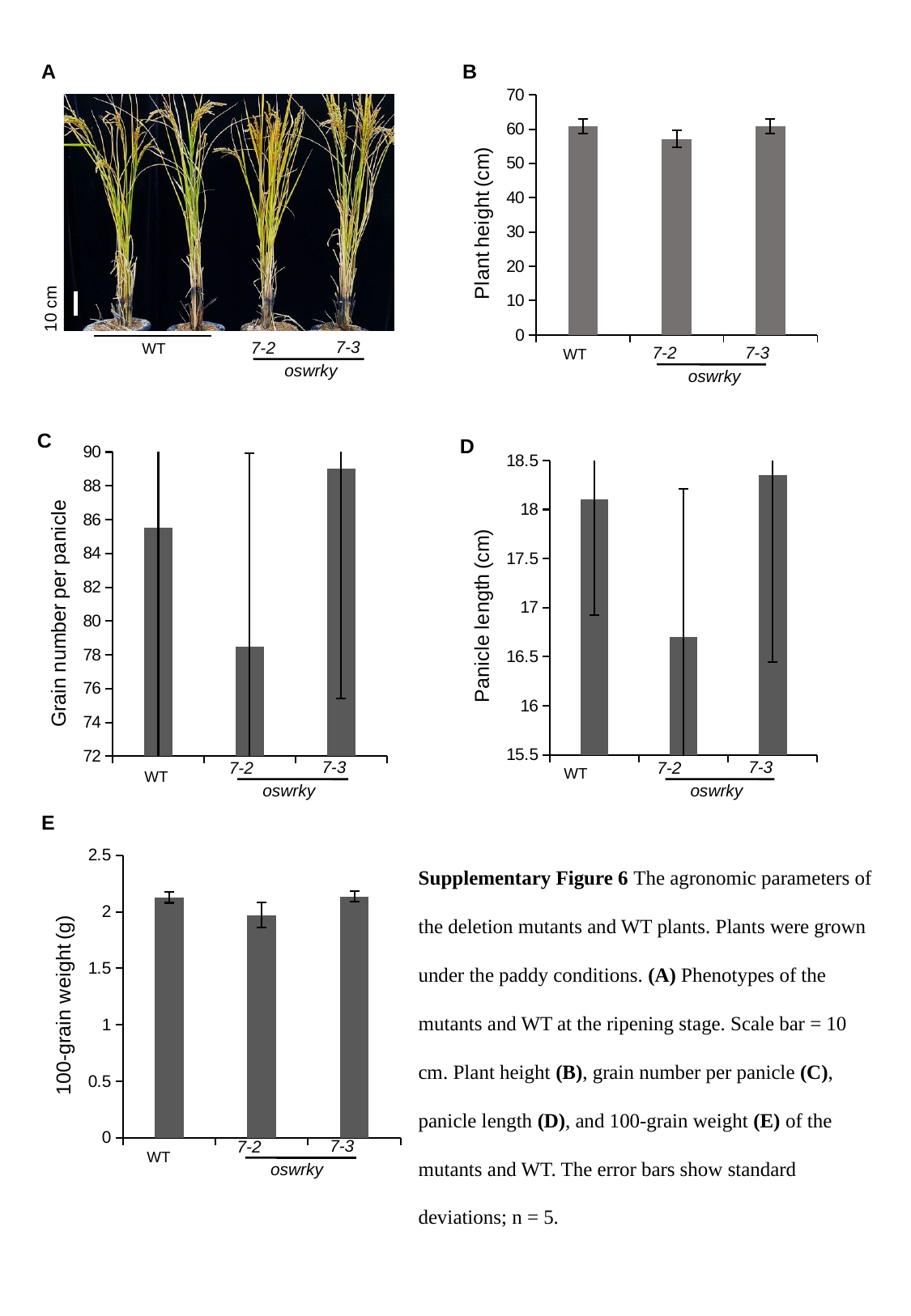

A
B
### Chart
| Category | |
|---|---|
| WT | 60.857142857142854 |
| CR-11 | 57.142857142857146 |
| CR-17 | 60.857142857142854 |
10 cm
7-3
7-2
oswrky
WT
7-3
7-2
WT
oswrky
C
D
### Chart
| Category | |
|---|---|
| WT | 85.5 |
| CR-11 | 78.5 |
| CR-17 | 89.0 |7-3
7-2
oswrky
WT
### Chart
| Category | |
|---|---|
| WT | 18.1 |
| CR-11 | 16.7 |
| CR-17 | 18.35 |7-3
7-2
oswrky
WT
E
Supplementary Figure 6 The agronomic parameters of the deletion mutants and WT plants. Plants were grown under the paddy conditions. (A) Phenotypes of the mutants and WT at the ripening stage. Scale bar = 10 cm. Plant height (B), grain number per panicle (C), panicle length (D), and 100-grain weight (E) of the mutants and WT. The error bars show standard deviations; n = 5.
### Chart
| Category | |
|---|---|
| WT | 2.128 |
| CR-11 | 1.97 |
| CR-17 | 2.136 |7-3
7-2
oswrky
WT

## Slide 8
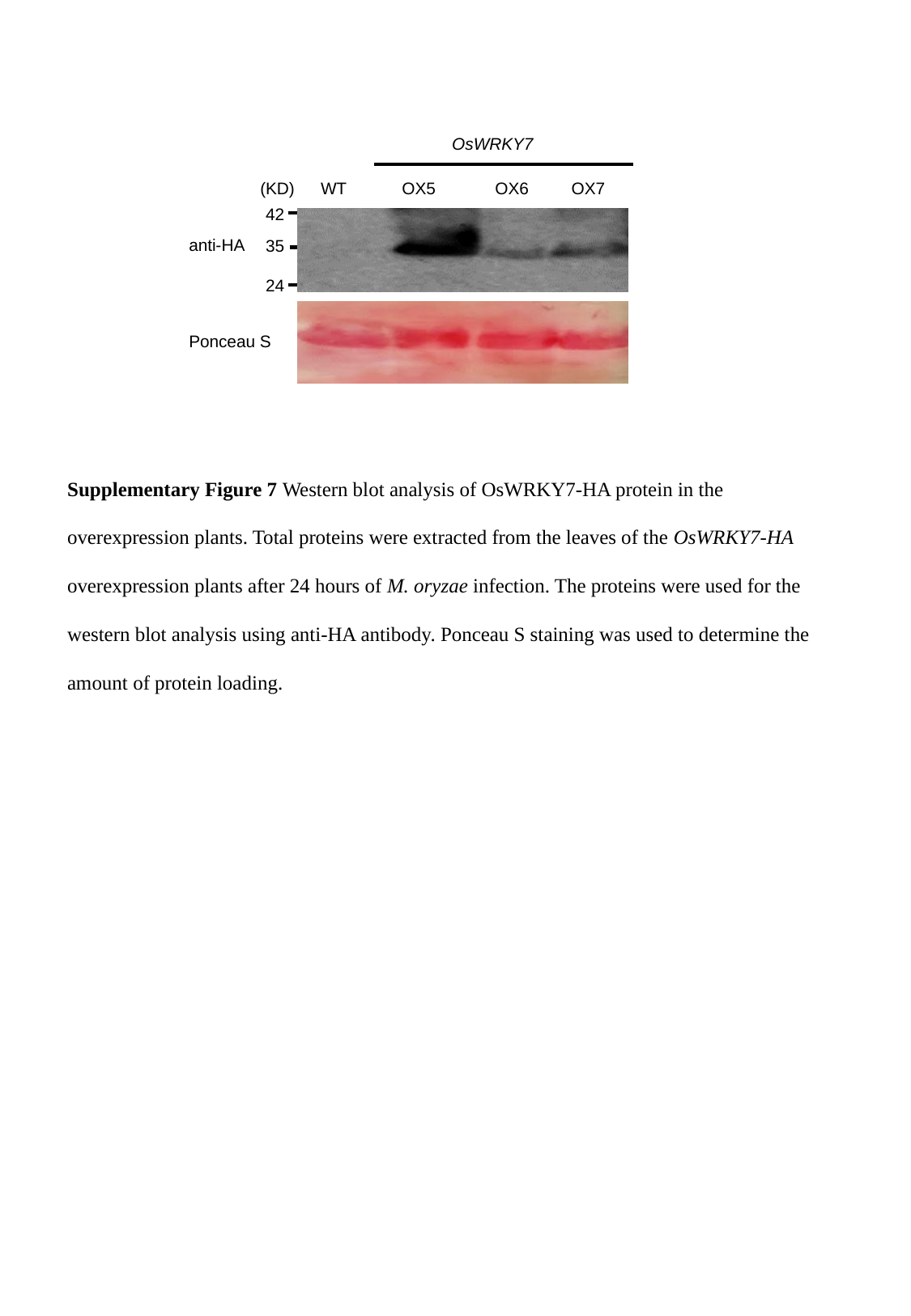

OsWRKY7
WT
OX5
OX6
OX7
(KD)
42
anti-HA
24
Ponceau S
35
Supplementary Figure 7 Western blot analysis of OsWRKY7-HA protein in the overexpression plants. Total proteins were extracted from the leaves of the OsWRKY7-HA overexpression plants after 24 hours of M. oryzae infection. The proteins were used for the western blot analysis using anti-HA antibody. Ponceau S staining was used to determine the amount of protein loading.

## Slide 9
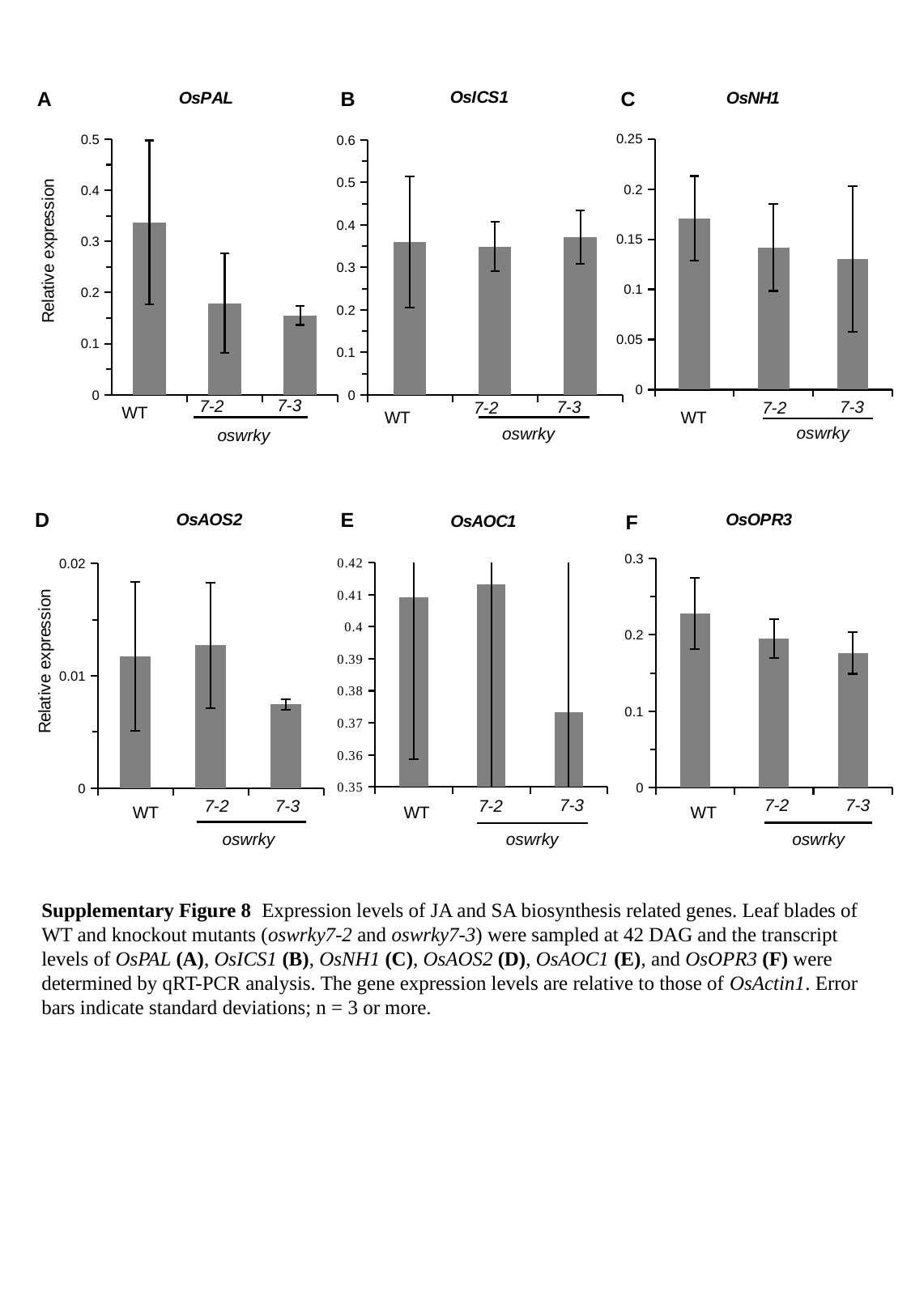

C
A
B
### Chart: OsNH1
| Category | |
|---|---|
| WT | 0.17071620896624487 |
| oswrky7-1 | 0.14167065803255563 |
| oswrky7-2 | 0.12996777765200565 |
### Chart: OsPAL
| Category | |
|---|---|
| WT | 0.33724357984487974 |
| oswrky7-1 | 0.17868022787708432 |
| oswrky7-2 | 0.15494131714405174 |
### Chart: OsICS1
| Category | |
|---|---|
| WT | 0.3598370711285989 |
| oswrky7-1 | 0.34915958638583994 |
| oswrky7-2 | 0.3706686468445092 |WT
WT
### Chart: OsOPR3
| Category | |
|---|---|
| WT | 0.22765286406215587 |
| oswrky7-1 | 0.19481262928993573 |
| oswrky7-2 | 0.17638287052916518 |
### Chart: OsAOC1
| Category | |
|---|---|
| WT | 0.4092461430658104 |
| oswrky7-1 | 0.4130764608114018 |
| oswrky7-2 | 0.37323508820005225 |
### Chart: OsAOS2
| Category | |
|---|---|
| WT | 0.011711096652577689 |
| oswrky7-1 | 0.01270619773703271 |
| oswrky7-2 | 0.007443153641673181 |WT
WT
WT
7-3
7-2
oswrky
7-3
7-2
oswrky
7-3
7-2
oswrky
WT
D
E
F
7-3
7-2
oswrky
7-3
7-2
oswrky
7-3
7-2
oswrky
Supplementary Figure 8 Expression levels of JA and SA biosynthesis related genes. Leaf blades of WT and knockout mutants (oswrky7-2 and oswrky7-3) were sampled at 42 DAG and the transcript levels of OsPAL (A), OsICS1 (B), OsNH1 (C), OsAOS2 (D), OsAOC1 (E), and OsOPR3 (F) were determined by qRT-PCR analysis. The gene expression levels are relative to those of OsActin1. Error bars indicate standard deviations; n = 3 or more.

## Slide 10
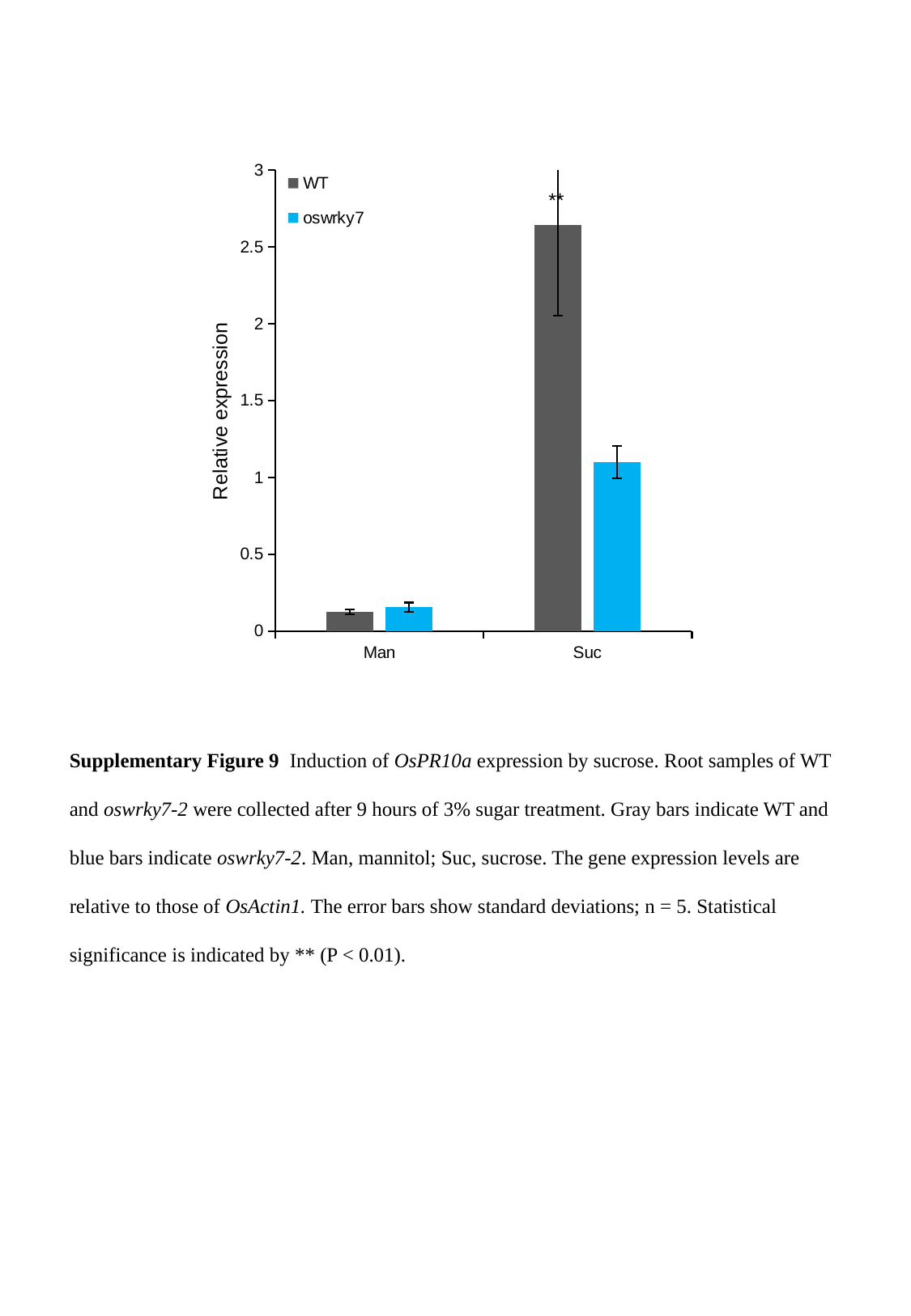

### Chart
| Category | WT | oswrky7 |
|---|---|---|
| Man | 0.12477934201033063 | 0.15553134903128857 |
| Suc | 2.6393264944923813 | 1.0999914815545668 |**
Supplementary Figure 9 Induction of OsPR10a expression by sucrose. Root samples of WT and oswrky7-2 were collected after 9 hours of 3% sugar treatment. Gray bars indicate WT and blue bars indicate oswrky7-2. Man, mannitol; Suc, sucrose. The gene expression levels are relative to those of OsActin1. The error bars show standard deviations; n = 5. Statistical significance is indicated by ** (P < 0.01).

## Slide 11
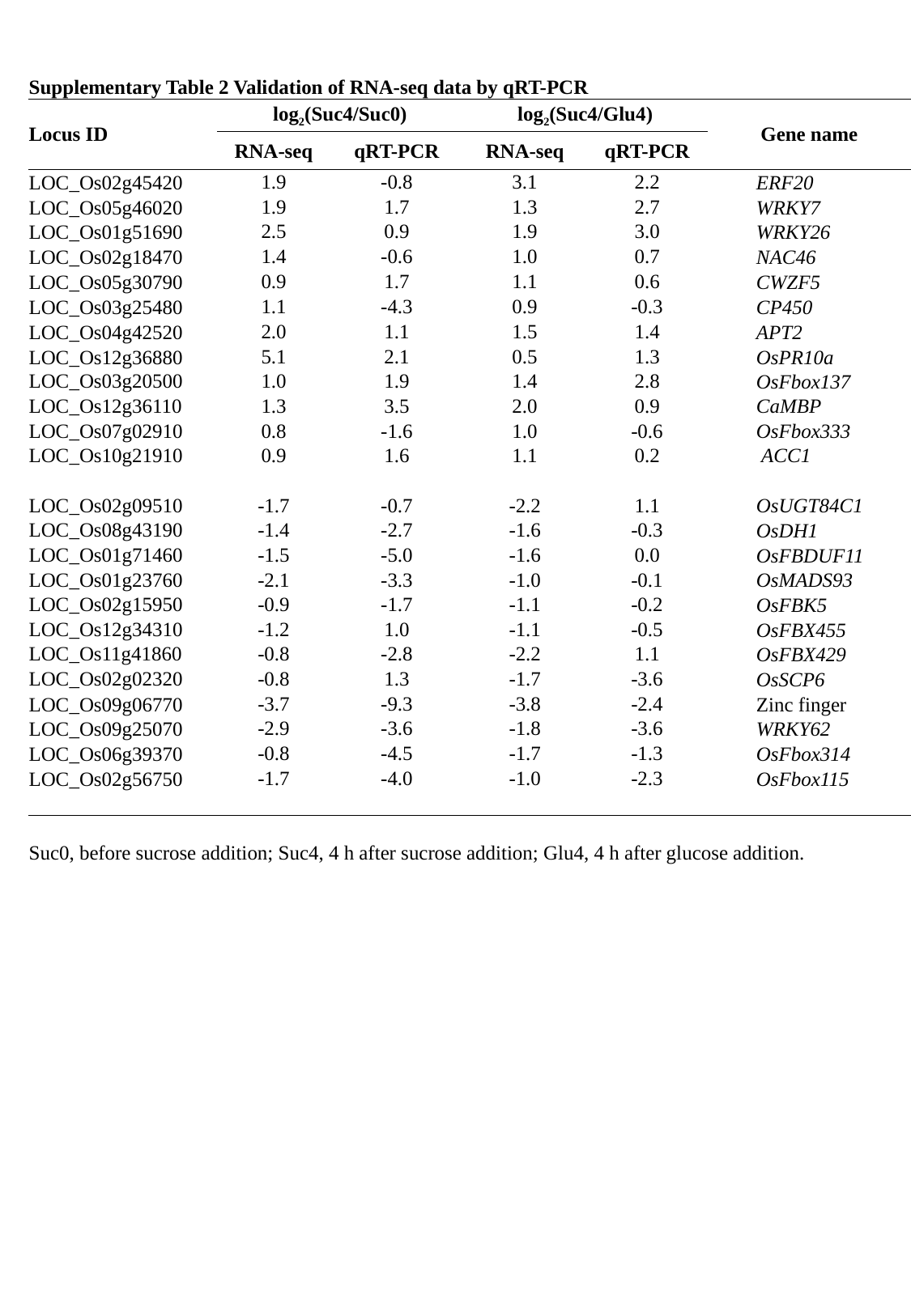

| Supplementary Table 2 Validation of RNA-seq data by qRT-PCR | | | | | |
| --- | --- | --- | --- | --- | --- |
| Locus ID | log2(Suc4/Suc0) | | log2(Suc4/Glu4) | | Gene name |
| | RNA-seq | qRT-PCR | RNA-seq | qRT-PCR | |
| LOC\_Os02g45420 | 1.9 | -0.8 | 3.1 | 2.2 | ERF20 |
| LOC\_Os05g46020 | 1.9 | 1.7 | 1.3 | 2.7 | WRKY7 |
| LOC\_Os01g51690 | 2.5 | 0.9 | 1.9 | 3.0 | WRKY26 |
| LOC\_Os02g18470 | 1.4 | -0.6 | 1.0 | 0.7 | NAC46 |
| LOC\_Os05g30790 | 0.9 | 1.7 | 1.1 | 0.6 | CWZF5 |
| LOC\_Os03g25480 | 1.1 | -4.3 | 0.9 | -0.3 | CP450 |
| LOC\_Os04g42520 | 2.0 | 1.1 | 1.5 | 1.4 | APT2 |
| LOC\_Os12g36880 | 5.1 | 2.1 | 0.5 | 1.3 | OsPR10a |
| LOC\_Os03g20500 | 1.0 | 1.9 | 1.4 | 2.8 | OsFbox137 |
| LOC\_Os12g36110 | 1.3 | 3.5 | 2.0 | 0.9 | CaMBP |
| LOC\_Os07g02910 | 0.8 | -1.6 | 1.0 | -0.6 | OsFbox333 |
| LOC\_Os10g21910 | 0.9 | 1.6 | 1.1 | 0.2 | ACC1 |
| | | | | | |
| LOC\_Os02g09510 | -1.7 | -0.7 | -2.2 | 1.1 | OsUGT84C1 |
| LOC\_Os08g43190 | -1.4 | -2.7 | -1.6 | -0.3 | OsDH1 |
| LOC\_Os01g71460 | -1.5 | -5.0 | -1.6 | 0.0 | OsFBDUF11 |
| LOC\_Os01g23760 | -2.1 | -3.3 | -1.0 | -0.1 | OsMADS93 |
| LOC\_Os02g15950 | -0.9 | -1.7 | -1.1 | -0.2 | OsFBK5 |
| LOC\_Os12g34310 | -1.2 | 1.0 | -1.1 | -0.5 | OsFBX455 |
| LOC\_Os11g41860 | -0.8 | -2.8 | -2.2 | 1.1 | OsFBX429 |
| LOC\_Os02g02320 | -0.8 | 1.3 | -1.7 | -3.6 | OsSCP6 |
| LOC\_Os09g06770 | -3.7 | -9.3 | -3.8 | -2.4 | Zinc finger |
| LOC\_Os09g25070 | -2.9 | -3.6 | -1.8 | -3.6 | WRKY62 |
| LOC\_Os06g39370 | -0.8 | -4.5 | -1.7 | -1.3 | OsFbox314 |
| LOC\_Os02g56750 | -1.7 | -4.0 | -1.0 | -2.3 | OsFbox115 |
| | | | | | |
| Suc0, before sucrose addition; Suc4, 4 h after sucrose addition; Glu4, 4 h after glucose addition. | | | | | |
